# Caveolin-1, a Key Mediator Across Multiple Pathways in Glioblastoma and an Independent Negative Biomarker of Patient Survival

**DOI:** 10.3389/fonc.2021.701933

**Published:** 2021-08-20

**Authors:** Chiara Moriconi, Prospero Civita, Catia Neto, Geoffrey J. Pilkington, Mark Gumbleton

**Affiliations:** ^1^School of Pharmacy and Pharmaceutical Sciences, College of Biomedical and Life Sciences, Cardiff University, Cardiff, United Kingdom; ^2^Department of Pathology and Cell Biology, Columbia University, New York Presbyterian Hospital, New York, NY, United States; ^3^Brain Tumour Research Centre, School of Pharmacy & Biomedical Sciences, University of Portsmouth, Portsmouth, United Kingdom; ^4^Department of Basic and Clinical Neuroscience, Division of Neuroscience, Institute of Psychiatry & Neurology, King’s College London, London, United Kingdom

**Keywords:** glioblastoma, caveolin-1, prognostic, survival, gender, TCGA, CGGA, invasion

## Abstract

Glioblastoma (GB) remains an aggressive malignancy with an extremely poor prognosis. Discovering new candidate drug targets for GB remains an unmet medical need. Caveolin-1 (Cav-1) has been shown to act variously as both a tumour suppressor and tumour promoter in many cancers. The implications of Cav-1 expression in GB remains poorly understood. Using clinical and genomic databases we examined the relationship between tumour Cav-1 gene expression (including its spatial distribution) and clinical pathological parameters of the GB tumour and survival probability in a TCGA cohort (n=155) and CGGA cohort (n=220) of GB patients. High expression of Cav-1 represented a significant independent predictor of shortened survival (HR = 2.985, 5.1 *vs* 14.9 months) with a greater statistically significant impact in female patients and in the Proneural and Mesenchymal GB subtypes. High Cav-1 expression correlated with other factors associated with poor prognosis: IDH w/t status, high histological tumour grade and low KPS score. A total of 4879 differentially expressed genes (DEGs) in the GB tumour were found to correlate with Cav-1 expression (either positively or negatively). Pathway enrichment analysis highlighted an over-representation of these DEGs to certain biological pathways. Focusing on those that lie within a framework of epithelial to mesenchymal transition and tumour cell migration and invasion we identified 27 of these DEGs. We then examined the prognostic value of Cav-1 when used in combination with any of these 27 genes and identified a subset of combinations (with Cav-1) indicative of co-operative synergistic mechanisms of action. Overall, the work has confirmed Cav-1 can serve as an independent prognostic marker in GB, but also augment prognosis when used in combination with a panel of biomarkers or clinicopathologic parameters. Moreover, Cav-1 appears to be linked to many signalling entities within the GB tumour and as such this work begins to substantiate Cav-1 or its associated signalling partners as candidate target for GB new drug discovery.

## Introduction

In adults, gliomas account for the majority of all primary malignant brain tumours, with over 50% of all gliomas constituted by the grade IV astrocytoma, glioblastoma multiforme (GB) (1). GB is one of the most aggressive tumours in humans. By the time of diagnosis, its highly invasive character often limits success in the total surgical resection of the tumour. GB displays a high level of angiogenesis and an ability to resist apoptosis upon exposure to chemo-/radio-therapies. It is a tumour prone to recurrence, with 5-year survival rates of no more than 5% (2) and which have not notably improved over the last three decades (3). Effective medical treatment for GB is a major unmet oncology need that will benefit from identification of robust predictive biomarkers that stratify patients at “high” or “low” risk of disease progression, and by the description of clinically meaningful molecular markers relevant for novel targeted therapies.

Recent genome-wide profiling studies such as the Cancer Genome Atlas (TCGA) and Rembrandt projects have helped to clarify the role of genomic alterations in the pathogenesis of GB (3, 4) and in stratifying patients based on specific molecular genotypes (5). New genome-wide profiling databases, such as the Chinese Glioma Genome Atlas (CGGA) have been developed in recent years. This study, and others (6–8), based on bulk tumour or single-cell sequencing have identified molecular markers (9) such as, methylation status of MGMT promoter and w/t IDH-1 status that have been widely explored as prognostic biomarkers for therapy responsiveness (10, 11). Such markers and clinical variables as patient age, Karnofsky performance score (KPS) and extent of resection are used as predictors of survival (12). In 2016, the World Health Organization (WHO) updated the classification of central nervous system (CNS) tumours, combining molecular parameters and histology (13). Aligned to this concept the Ivy Glioblastoma Atlas Project (14) (a collaborative network among bioinformaticians, physician and pathologists) presents an extensive database of GB histological sections along with the respective tumour tissue genetic alterations and gene expression profile with the aim to describe heterogeneity of the GB at the molecular and cellular levels.

Caveolin-1 (Cav-1) is a major structural and functional protein of caveolae membrane domains involved in the compartmentalisation and orchestration of cell signalling activity. It is a regulator of multiple signal transduction events and cytoskeletal dynamics, able to interact often in a cell- and context specific-manner with multiple cell signalling partners modifying downstream actions (15–17). At least in preclinical models Cav-1 is shown to modulate several signalling pathways to promote and/or suppress the malignant phenotype (18). For example, Cav-1 has been shown to facilitate both ERK and AKT signalling in cancer cells derived from prostate (19) and colon (20), and is associated with promoting cell invasion, proliferation, angiogenesis and multi-drug resistance. The role of Cav-1 in malignancy is however both complex and multifaceted with both tumour suppressor and oncogenic properties. For example, elevated levels of Cav-1 in clinical tumour tissue from prostate (19), bladder (21), kidney (22) and multiple myeloma (23) is unequivocally linked with metastasis and poor prognosis. Meanwhile in carcinomas of the breast (24), colon (25) and lung (26) both the loss and gain of Cav-1 have been associated with tumour progression. The understanding of Cav-1 biology in GB is similarly controversial, with some reports suggesting Cav-1 to be a tumour suppressor (27–29) and others supporting the oncogenic function (30). A few studies have reported positive correlations between Cav-1 expression and increased tumour histological grade (31, 32). Cav-1 expression has also been reported to independently predict shorter survival in oligodendrogliomas (33), although this finding is equivocal (31). Most recently, Cav-1 has been identified as marker in glioma, promoting invasion by modulation of matrix-degrading enzyme (34) with unfavourable outcomes in glioma patients (35).

This current work tests the hypothesis that Cav-1 serves as an independent prognostic marker in high grade glioma, specifically in GB. Using clinical and genomic databases (TCGA, CGGA and IVY) we examined the relationship between Cav-1 gene expression (including Cav-1 protein spatial distribution within the tumour) and known clinical pathological parameters of the GB tumour and the survival probability in a cohort GB patients. We then used the TCGA database to further explore the predictive prognostic capacity in GB of Cav-1 when used in combination with other molecular markers. This involved exploration of the genes whose expression within the GB tumour co-correlated (positively or negatively) with Cav-1; we identified 4879 such genes (‘differentially expressed genes’; DEGs). Focusing on those that lie within a framework of known mechanisms of epithelial to mesenchymal transition (EMT) and tumour cell migration and invasion we identified 27 DEGs. We then examined the prognostic value of Cav-1 when used in combination with any of these genes and identified a subset of combinations (with Cav-1) indicative of co-operative synergistic mechanisms of action.

## Materials and Methods

### Bioinformatic and Statistical Analysis

#### TCGA and CGGA Databases and Survival Analysis

The human glioblastoma analysis was first performed on the TCGA dataset, available as ‘Tumour Glioblastoma - TCGA - MAS 5.0 - u133a’ (Network, C. G. A. R 2008) in the R project 3.5.0, and source data are available at https://www.cbioportal.org. The TCGA database used comprises information about 540 patients, including 85 samples sub-classified in classical (n = 17), mesenchymal (n = 27), neural (n = 17) and proneural (n = 24) GB. While,for CGGA datasets, data were downloaded from http://www.cgga.org.cn. The mRNA expression and suvival analysis was performed only on primary or “*de novo*” GB cohort (n=220). The survival statistical analysis was performed using R2 webtool (36) and Survminer package (37). The Survminer package provides Kaplan-Meyer plots based on a Log Rank Scale p-value for the comparison of the subgroups. The optimal cut off was calculated by maximally selected rank statistics using Maxstat R-package (38).

### Preclinical Data Statistical Analysis

Preclinical data was analysed using T-test (unpaired) for two groups and by more than two groups using one-way ANOVA with Tukey’s test (comparisons across all groups). Multivariate analysis was carried out by COX regression using Enter and Forward function with covariates marker considered as categorical in each model.

### Functional Enrichment Analysis

Functional enrichment analysis of the DEGs between tumour compartments and control tissue and between different tumour compartments was performed using FunRich (39) analysis tool. Functional enrichment was carried out for Biological process using Entrez ID genes nomenclature.

### IVY Glioblastoma Atlas Project

Gene expression, clinical and genomic data on primary diagnosis of GBs and their donors were collected from the Ivy Glioblastoma Atlas Project. Z-score normalized expression values of Cav-1 was downloaded from the Anatomic Structures RNA-Sequencing data set [Available in: glioblastoma.alleninstitute.org/rnaseq/search/index.html]. These are given as fragments per kilobase per million (FPKM), and further adjusted with TbT normalization (by scaling each sample based on the summed expression of all genes that are not differentially expressed). Gene expression data was obtained by the RNA-seq technique, applied to the seven GB histological structures that were isolated by laser capture microdissection (LCM) in each histological section of tumour blocks: Leading Edge (LE), Infiltrating Tumour (IT), Cellular Tumour (CT), Perinecrotic Zone (PN), Pseudopalisading Cells (PS) around Necrosis, Hyperplastic Blood Vessels (HBVs) in Cellular Tumour and Microvascular Proliferation (MpVs). In particular, the vasculature (MpVs, HBVs) and hypoxic (PN) regions where validated by the enrichment for endothelial and hypoxic markers (40), also subtyping 90 nonvascular regions using the 4 gene expression signatures defined by TCGA.

### Immunohistochemistry Reaction of Caveolin-1

Protein expression in high grade glioma (GB) tissues and normal brain cortex tissues was determined using the Human Protein Atlas database (2018 version, www.proteinatlas.org/). The Human Protein Atlas is a database of immunohistochemistry (IHC)-based protein expression profiles in normal tissue, cancer and cell lines (41). IHC images of Cav-1 protein expression in clinical specimens of patients with high grade glioma and normal brain cortex tissue refer to the antibody CAB003791.

### Cell Culture and CRISPR Transfection

The U87MG cell line was maintained in normal culture medium, Dulbecco’s Modified Eagle Medium (DMEM), 10% Fetal Bovine Serum (FBS), 1% Penicillin-Streptomycin (PS) (Life Technologies, Fisher Scientific, Paisley, UK). Plasmids U6gRNA-Cas9-2A-GFP (Sigma-Aldrich, Gillingham, UK) were used to achieve CRISPR knockdown of Cav-1. They were replicated into Max Efficiency DH5a Competent Cells (Life Technologies). Cell sorting for GFP-positive cells isolated, single positive cloned and the surviving colonies were analysed for the expression of Cav-1 *via* Western Blot.

### Proliferation Assay

Replicates of 5000 cells/cm^2^ were seeded in multiple wells and maintained in normal culture medium. At discreet points wells were supplied with one volume of CyQuant® Direct Cell Proliferation Assay Kit (Invitrogen, Life Technologies, Paisley, UK) for the staining of DNA content and indirect quantification of cell proliferation. Fluorescence data were plotted, and doubling time was calculated on the viability data corresponding to the log phase of each plot.

### Colony Assay

6-well plates were seeded with 500 cells per well in normal culture medium and cells were grown for 7 days. Staining with Crystal Violet allowed the visualization of the newly formed colonies. Plates were imaged on a ChemiDoc XRS+ (BIORAD, Hertfordshire, UK) with a modified copper staining protocol. FIJI plugin Cell Counter was used to quantify the space occupied by colonies (42).

### 3D Invasion Assay

A 3D invasion assay was performed as described in Vinci et al. (43). 1000 cells were seeded in multiple wells of ultra-low adherence round bottom 96-well plates with normal culture medium. After a gentle centrifugation (300g- 1 min) the plates were incubated at 37°C 5% pCO2 for the formation of tight aggregates. After four days half of the medium was replaced with growth factor reduced Matrigel ™ (Corning) on ice. One hour was allowed on ice for the medium and the Matrigel to diffuse, after which gelification was achieved in incubator. Images were captured at T0 and every 24 hours. The analysis of the 2D greyscale images was obtained by the customizable FIJI script, INSIDIA (44).

### Statistical Analysis for *In Vitro* Test

Statistical analysis for the *in vitro* experiments was performed using GraphPad (GraphPad Prism version 7.00, GraphPad Software, La Jolla California USA, www.graphpad.com). Student’s T-Test (unpaired two-tailed) was employed for comparisons between two experimental groups (statistical significance set at P < 0.05). One-way ANOVA statistical analysis followed by an appropriate *post hoc* test was applied for comparisons involving more than two experimental groups. For equal group sizes a Tukey’s multiple comparison test has been performed. For unequal groups sizes a Tukey-Kramer test was used. For the comparison of multiple groups to a single control treatment, a Dunnett test was chosen.

## Results

### Survival Analysis: Cav-1 Is an Independent Negative Prognostic Marker for GB Patients

**Figure 1:**
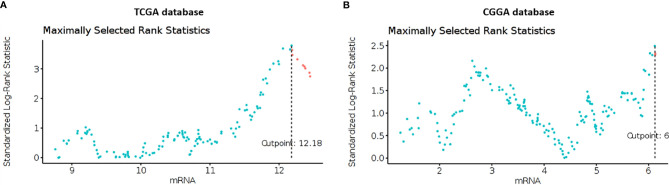
**(A, B)** Maximally selected rank statistics plot of Cav-1 expression (mRNA) for GB patients determined from the TCGA **(A)** and CGGA **(B)** databases. The plots show the cut-point (mRNA level) that corresponds to the most optimal (statistically significant) discrimination with respect to outcome (i.e. survival). Light blue dots represent patients who have low expression of Cav-1, and pink dots represent patients who have high expression of Cav-1.

Using clinical and genomic data from the TCGA and CGGA (8) databases we investigated if Cav-1 serves as an independent prognostic marker in GB patients, we calculated the optimal expression cut-point for survival using maximally selected log-rank statistic. Using data from TCGA database, the standardised method yielded 12.18 as the best cut-point which refers to the mRNA expression level (**Figure 1A**) discriminating between two groups of patients with respect to overall survival. We then extended the analysis to the CGGA database (n=220 patients) which yielded 6.12 as a value of mRNA that dichotomized the GB cohort in “high” and “low” expression of Cav-1 (**Figure 1B**).

**Figure 2:**
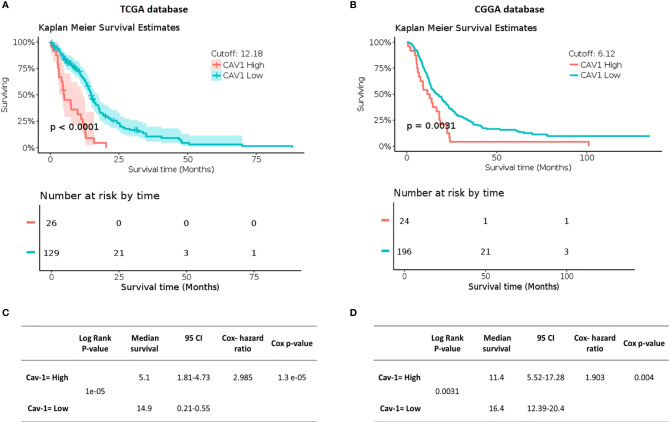
Impact of Cav-1 expression on GB patient overall survival in TCGA **(A, C)** and CGGA databases **(B, D)**. **(A, B)** show respectively (TCGA and CGGA) Kaplan Meier survival curves for GB patients where the tumours displayed either high or low expression of Cav-1 (separated by a mRNA cut point of 12.18 and 6.12 respectively). The survival curves are supported by the corresponding patient numbers at risk by each timepoint (shown below the respective KM curve). **(C, D)** show respectively, (TCGA and CGGA) Univariate Cox regression analysis for Cav-1 expression and the associated GB cohort including the 95% confidence interval (CI), median survival in months. Cox Hazard ratio and the p-value.

Using clinical information from TCGA and CGGA databases we then generated Kaplan-Meier survival outcomes for GB patients (**Figures 2A, B**). In the TCGA database, patients with high expression of Cav-1 had a mean survival of 5.1 months (95% CI, 1.81-4.73 months) compared with 14.9 months (95% CI, 0.21-0.55 months) for patients with a low expression of the gene (**Figure 2A**). For CGNA database (cut point 6.12) patients with high expression of Cav-1 had a mean survival of 11.4 months (95% CI, 5.52-17.28 months) compared with a mean survival of 16.4 months (95% CI, 12.39-20.5 months) in patients with ‘low’ Cav-1 expression (**Figure 2B**). The univariate Cox proportional-hazards models analysis (**Figures 2C, D**) show high expression of Cav-1 to be a significant independent predictor of shortened survival with a hazard ratio (HR) of 2.985 (Cox p-value = 0.0000013) in the TCGA database (**Figure 2C**) and 1.903 (Cox p-value = 0.004) in the CGGA database, confirming a strong relationship between high Cav-1 expression and poor prognosis in both cohorts considered.

**Figure 3:**
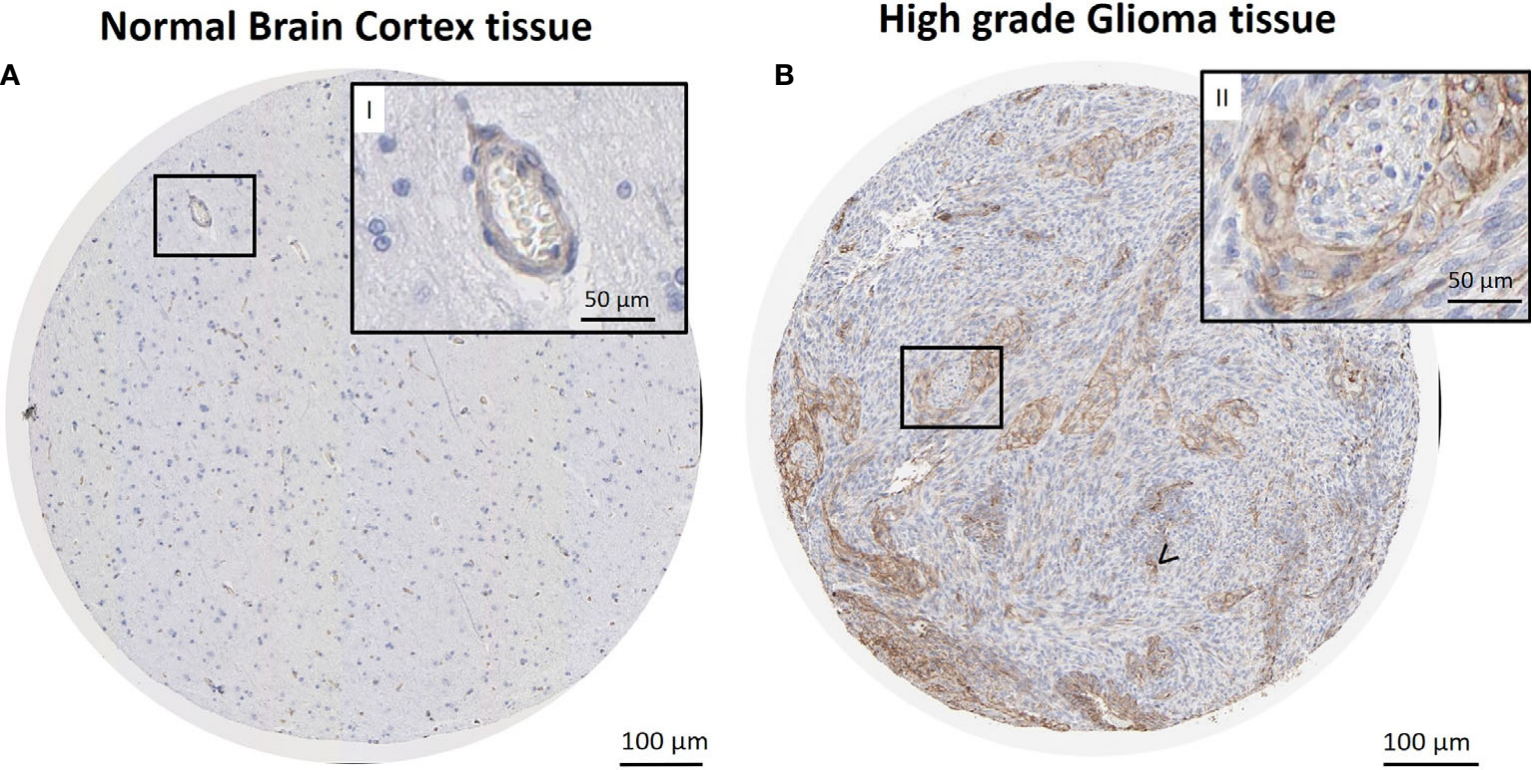
Immunohistochemical reaction for Cav-1 in normal brain cortex tissue (control) and GB tissue. Sections retrieved from The Human Protein Atlas project. Representative sections of high-grade glioma show a strong cytoplasmatic/membranous positivity of Cav-1 in tumour cells **(B)**, also in higher magnification **(B, II)**. Strong positivity is also observed in microvasculature areas **(B)**, arrow <). No reaction is observed in glial cells of normal brain cortex tissue **(A)**. Lower expression of Cav-1 in endothelial cells and capillaries was also evident in the normal tissue control material **(A, II)**. Scale bars: **(A, B)** 100 µm; (**I-II**, higher magnification) 50 µm.

Cav-1 mRNA expression in GB (n =155) was also evaluated using the online Human Protein Atlas database (HPA, www.proteinatlas.org) in order to compare the quantitative RNA-seq data with the spatial expression data for corresponding protein levels. Immunohistochemical analysis (**Figure 3**) showed a strong cytoplasmatic/membranous positivity (75%) of Cav-1 within tumour cells (**Figure 3B**, magnification II) in tissue sections of high grade glioma compared to control (non-tumour) tissue from brain cortex, where little to no Cav-1 staining in glial cell populations was evident (**Figure 3A**). Endothelium did show positive Cav-1 staining, which was of a strong intensity in the tumour sections (**Figure 3B**, arrow) but of lower intensity (25%) within endothelial cells of non-tumour brain cortex tissue (**Figure 3B**, magnification I) in normal brain cortex tissue.

### Survival Analysis: Gender and Cav-1 Expression

The univariate analysis of the TCGA dataset of GB patients (n=155) revealed no difference in survival by gender (median survival: male 378 days *vs* female 399 days; (**Supplementary Figure S1A**). However, multivariate analysis, combining Cav-1 expression and gender did show a gender component on patient survival (**Supplementary Figure S1B**). Specifically, female patients expressing high tumour levels of Cav-1 displayed a significantly shorter median survival time compared to male patients expressing high levels of Cav-1 (median survival 90.5 days *vs* 320 days: HR=3.145, P=0.0000015). In contrast, there was no gender-based adverse outcome on survival in patients whose tumours expressed low levels of Cav-1, i.e. median survival (M) 427 days *vs* (F) 419 days (HR= 1.471, P=0.066).

### Correlation of Clinical Prognostic Indicators and Cav-1 Expression

In the TCGA dataset of GB patients we next analysed the relationship between Cav-1 expression and some commonly used markers that serve as clinical prognostic indicators and of relevance to classifying GB (12, 45). Firstly, we looked at MGMT, EGFR-vIII, PTEN and TP53 molecular status and found no significant correlation of these markers with Cav-1 expression (**Supplementary Figures S2A–D**).

**Figure 4:**
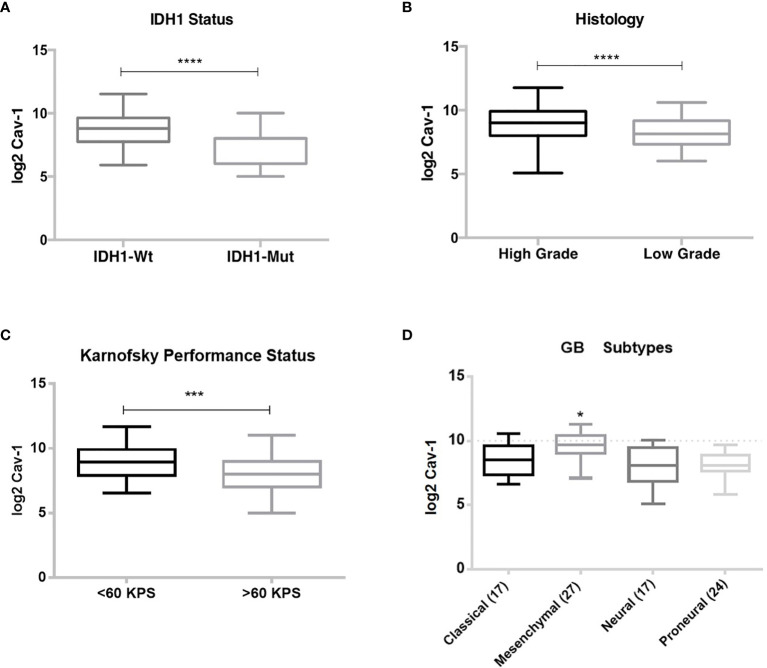
Correlation of Cav-1 tumour expression with prognostic indicators: **(A)** Isocitrate Dehydrogenase 1 (IDH1) status; **(B)** Histological grade, Low or High-grade; **(C)** Karnofsky performance status; **(D)** Verhaak et al. (5) classification. Boxes represent median (horizontal line) Cav-1 tumour expression (expressed as log2-fold) and 25th and 75th percentiles (error bars). Data were analysed using Student T-test (unpaired) for comparison of two groups where ***p < 0.0005: ****p < 0.0001. One-way ANOVA and Tukey’s test were used for multiple comparisons (GB subgroups) where *p < 0.0001.

Using the WHO classification for CNS tumours (13) we then analysed the GB dataset with respect to IDH1 status, where IDH1-wild type corresponds to primary or *de novo* GB, and the IDH-mutant corresponds to secondary or progressive GB. Here we found increased tumour expression of Cav-1 to be associated with the IDH-wild type patient cohort (P < 0.0001) (**Figure 4A**). Subgrouping by histological grade, high Cav-1 tumour expression was associated (P=0.0003) with high-grade GB (**Figure 4B**). Karnofsky Performance Scale (KPS) classifies patients in respect to their functional impairment and is used in assessing patient tolerability to treatment and patient prognosis. Perhaps not surprisingly we found a significantly greater tumour expression of Cav-1 in patients with a poor performance status (**Figure 4C**; <60 Karnofsky scale, P<0.0005) (46). Classifying GB subtypes according to Verhaak et al. (5), Cav-1 tumour expression was significantly (p < 0.0001) increased in the Mesenchymal GB subtype compared to all other subtypes (**Figure 4D**).

Cox-regression analyses was undertaken to determine the prognostic value of Cav-1 tumour levels as a covariate with IDH1 status (wild type or mutation), MGMT promoter methylation, EGFR-vIII amplification, PTEN and TP53 mutation (**Table 1A**). Therefore, the above markers together with Cav-1 were entered into the model. The analysis revealed that Cav-1 high and IDH-wt are both significantly influential prognostic predictor of survival with an HR of 2.88 (p<0.0001) and an HR of 6.45 (p<0.015) respectively. Of note, the composite co-variate (**Table 2B**) of ‘Cav-1 high and IDH-wt’ was a significant and powerful and influential prognostic with a HR of 11.4 (p<0.0001). Whereas markers such as PTEN-mutated and EGFR-amplified did not show meaningful multivariate analysis (**Table 1A**), when considered as a composite variable with Cav-1 they both were shown to be robust prognostic indicators (**Table 1B**).

**Table 1:** **(A, B)** Multivariate Cox proportional hazards model using Enter and Forward stepwise function including IDH-1, MGMT promoter, EGFRvIII, TP53, PTEN genes.

(A) Prognostic factors in the multivariate model	p-value	Hazard Ratio (HR)	95.0% CI for Hazard Ratio	
			Lower	Upper
Cav-1 (high *vs* low)	**0.001**	**2.867**	1.577	5.214
IDH-1 (wild type *vs* mutation)	**0.015**	**6.458**	1.430	29.170
MGMT promoter (methylated *vs* unmethylated)	0.203	0.721	0.436	1.192
PTEN (mutation *vs* wild type)	0.103	2.011	0.868	4.659
EGFRvIII (amplification *vs* not amplification)	0.657	1.126	0.668	1.896
TP53 (mutation *vs* wild type)	0.444	1.267	0.6912	2.324
**(B) Prognostic factors in the composite covariate model**	**p-value**	**Hazard Ratio (HR)**	**95.0% CI for Hazard Ratio**	
			**Lower**	**Upper**
Cav-1 “low”/IDH-1 mut		1		
Cav-1 “high”/IDH-1 wt	**<0.0001**	**11.480**	3.280	40.187
Cav-1 “low”/MGMT meth		1		
Cav-1 “high”/MGMT unmeth	0.454	0.825	0.498	1.366
Cav-1 “low”/PTEN wt		1		
Cav-1 “high”/PTEN mut	**0.031**	**4.773**	1.151	19.796
Cav-1 “low”/EGFR-vIII not ampl		1		
Cav-1 “high”/EGFR-vIII ampl	**0.005**	**3.527**	1.466	8.481
Cav-1 “low”/TP53 wt		1		
Cav-1 “high”/TP53 mut	0.195	1.971	0.706	5.502

HR, hazard ratio; CI 95%, Confidence interval; wt, wildtype; mut, mutated; ampl, amplification; unmeth, unmethylated; meth, methylated. Bold denotes significance of p < 0.0001.

**Table 2:** Correlation analysis in GB of Cav-1 and genes (identified from the pathway analysis) related to cell adhesion, ECM organisation and EMT pathways.

Gene Name	R-value	R-p value	Univariate poor prognosis (*section 2.7)	References
	Positive correlation with Cav-1			
PAI1	0.639	8.96E-60	✓	(47)
CD44	0.555	8.70E-42	✓	(48)
ITGB1	0.547	1.97E-40		(49)
UPAR	0.529	1.80E-37	✓	(47)
ITGA5	0.518	1.09E-35	✓	(28)
CTSB	0.488	3.00E-31	✓	(47)
UPA	0.482	1.83E-30	✓	(47)
TIMP1	0.428	1.45E-23	✓	(47)
CTSL	0.42	1.24E-22	✓	(47)
ITGA3	0.384	1.10E-18		(49)
ITGB5	0.37	2.12E-17	✓	(50)
TSP1	0.368	3.19E-17	✓	(51)
VIM	0.363	1.06E-16		(52)
ITGAV	0.33	7.12E-14		(50)
CTSS	0.301	1.30E-11		(53)
MMP1	0.301	1.42E-11	✓	(54)
MMP7	0.3	1.50E-11	✓	(55)
CTSD	0.296	3.02E-11	✓	(47)
MT1MMP	0.265	3.55E-09	✓	(47)
MMP9	0.226	7.30E-07	✓	(47)
MMP10	0.201	1.23E-05	✓	(55)
CTSH	0.158	0.0007410		(53)
CTSK	0.143	0.0024334		(56)
MMP3	0.128	0.0074362		(57)
TIMP3	0.115	0.0165875		(58)
ITGB3	0.1	0.0398341		(59)
MMP2	0.096	0.0494357	✓	(47)
	**Negative correlation with Cav-1**			
ECAD	-0.172	0.0002088		(52)

R value indicates the correlation value between Cav-1 and the selected genes; R-p value indicates the statistical significance. The data in **Table 2** for 27 genes are extracted from linked to **Supplementary xlsx Sheet** which include the 4879 genes correlating with Cav-1.

**Figure 5:**
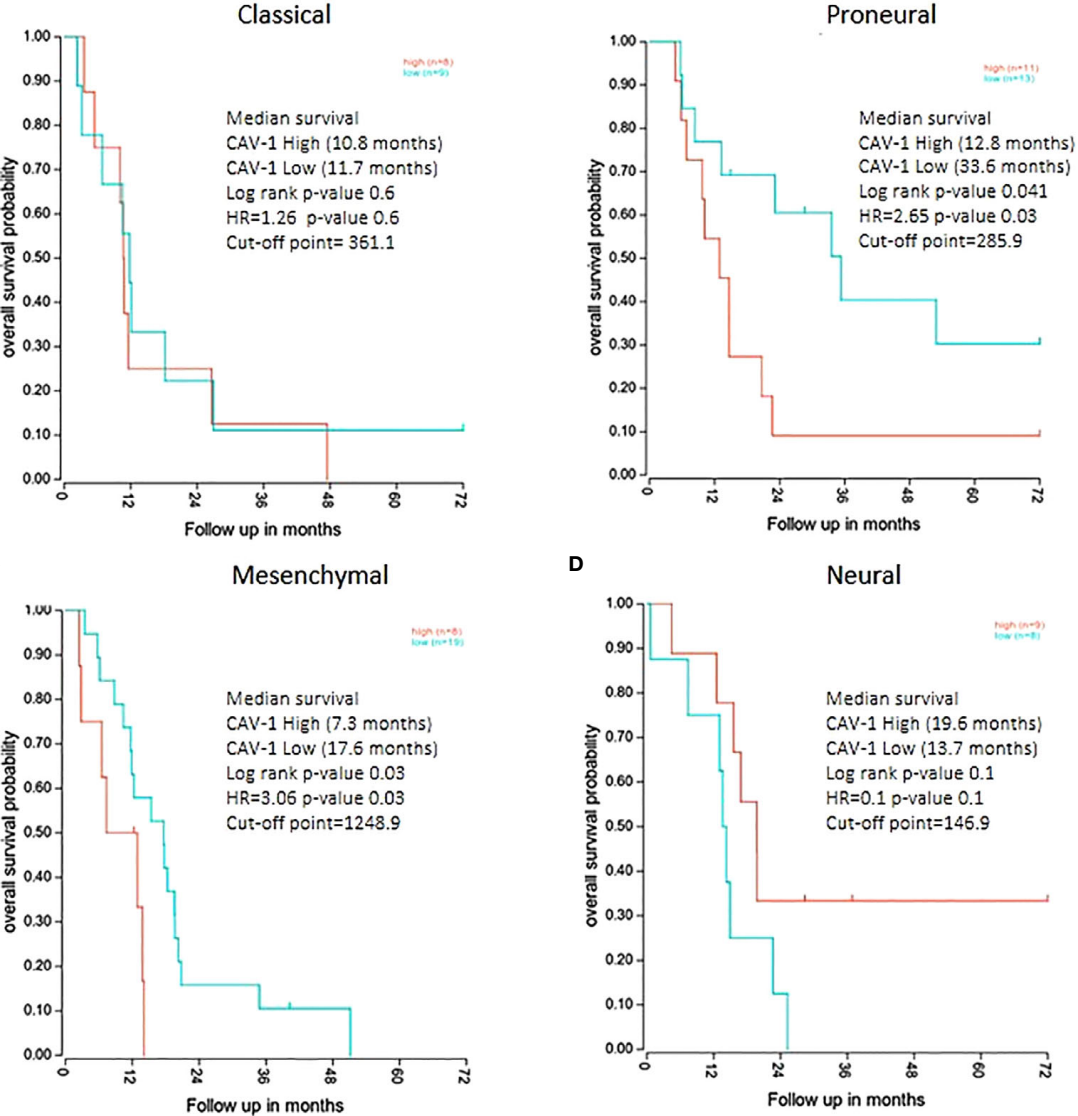
Kaplan Meier survival analysis of GB patients divided by molecular subtype groups with survival categorised by high and low tumour expression levels of Cav-1: **(A)** Classical (cut-point value=361.1), **(B)** Proneural (cut-point value=285.9), **(C)** Mesenchymal (cut-point value=1248.9), **(D)** Neural (cut-point value=146.9). For each curve the log rank p-value, median survival in months and Hazard Ratio value (HR) is reported. The plots show the cut-point (expression level) that corresponds to the most optimal (statistically significant) discrimination point with respect to outcome (i.e. survival).

We then examined the survival of patients by the tumour level of Cav-1 but across GB subtypes. High tumour expression of Cav-1 had a negative effect upon survival in both the Proneural (P=0.041) and Mesenchymal (P=0.035) subtypes. In the Proneural subtype (**Figure 5B**) high tumour Cav-1 levels were associated with a significantly shorter survival (high Cav-1 median survival of 12.8 months *vs* low Cav-1 of 33.6 months). Similarly, in the Mesenchymal subtype (high Cav-1 median survival of 7.3 months *vs* low Cav-1 of 17.6 months) (**Figure 5C**). No statistical difference was observed in respect to the tumour expression of Cav-1 and survival in either the Classical or Neural subtypes (**Figures 5A, D**, respectively).

### Cav-1 Shows Specific Distribution Patterns Within GB Tissue

**Figure 6:**
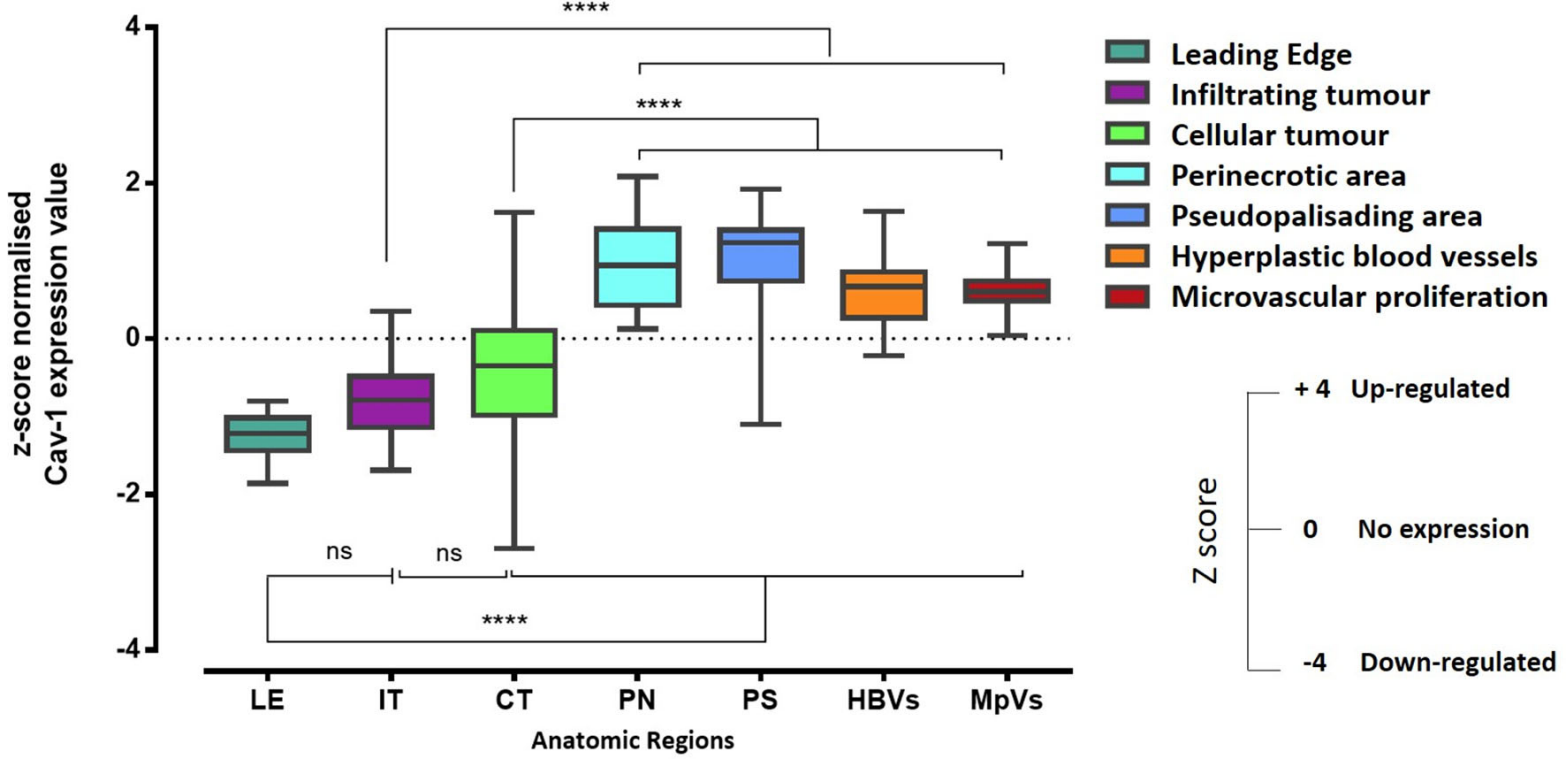
RNA-Seq expression z-score computed for Cav-1 differentially expressed within histological regions of the GB tumour (n-=41). The corresponding histological features are labelled as follows: HBVs, Hyperplastic blood vessels in cellular tumour; MpVs, Microvascular proliferation; PN, Pseudo-palisading cells around necrosis; IT, Infiltrating tumour; LE, Leading edge; CT, Cellular tumour. Error bars represent the SEM. One-way ANOVA and Tukey’s multiple comparison were used; ns, not significant; ****represents p < 0.0001.

We then analysed the regional expression of Cav-1 in 41 GB tissue blocks (41 patients) from the IVY Glioblastoma Atlas database (http://glioblastoma.alleninstitute.org), by means of z-score (expression value). Cav-1 appeared to be more highly expressed within hyperplastic blood vessels (HBVs), microvasculature proliferations (MpVs), peri-necrotic zone (PN) and pseudo-palisading cells around necrosis (PS) (**Figure 6**). In contrast, Cav-1 was less highly expressed in the following zones: leading-edge (LE), infiltrating tumour (IT) and in cellular tumour (CT; defined where tumour cells exceed normal cells ~100–500-fold) (**Figure 6**). The tumours of a subset of these patients’ (eight patients) were additionally analysed by in-situ hybridization (ISH) for Cav-1 (https://glioblastoma.alleninstitute.org/ish). The ISH analysis confirmed the regional expression of Cav-1 assessed by laser micro-dissection and RNA-seq methodologies (**Supplementary Figures S3A, B**).

### *In Vitro* CRISPR Cav-1 KO Shows Cav-1 Expression as Essential for U87 Tumorigenic and Invasive Abilities

**Figure 7:**
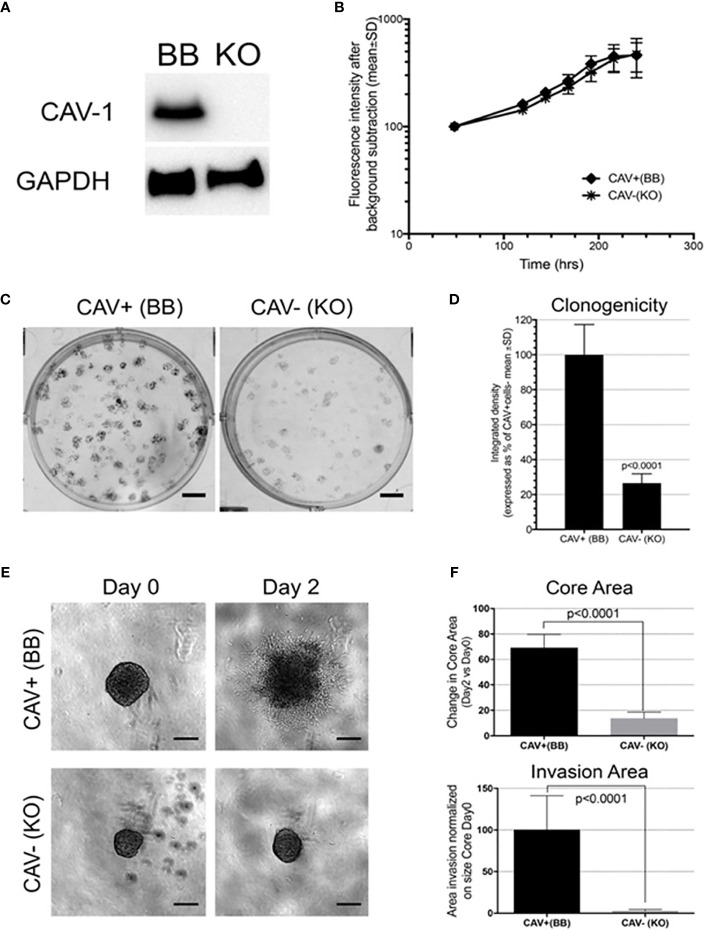
Cav-1 knockout (KO) and impact on the *in vitro* aggressiveness of the GB cell line U87: **(A)** Western blot for Cav-1 in the U87 cell line bearing genetic CRISPR deletion of Cav-1 (designated KO) compared to the Cav-1 positive control (designated BB). GAPDH was used as reference housekeeping gene. **(B)**; Proliferation curves of U87 Cav-1 +ve (BB) and U87 Cav-1 -ve (KO); **(C, D)** Clonogenicity assay with 7-day colony formation on agar U87 Cav-1 +ve (BB) *vs* Cav-1 -ve (KO) Representative images are shown in **(C)**. Scale bar 500 µm. Quantification is shown in **(D)**; **(E, F)** 3D *in-vitro* invasion assay. In 7E representative images of U87 Cav-1 +ve (BB) *vs* Cav-1 -ve (KO) spheroids embedded within Matrigel (Day 0) and the spheroid cell invasion after 2 days in Matrigel (Day 2). Scale bar 200 µm. In 7F quantification of the change (Day 2 *vs* Day 0) in the spheroid Core Area (top) and the spheroid area of the invasive edge (normalized to the perimeter of the core (bottom). This analysis undertaken as described previously using INSIDIA analysis software (44).

To directly assess the independent activity of Cav-1 in high grade glioma cells we modulated Cav-1 gene expression by the CRISPR system in the Cav-1 expressing U87 glioma cell line. Deletion of Cav-1 was detected at the protein level (**Figure 7A**). While the CRISPR knockout did not determine any change in the proliferation rate of the U87 (**Figure 7B**), the cells lacking Cav-1 (KO) displayed reduced colony formation (**Figures 7C, D**) as well as reduced invasion in a 3D matrix (**Figures 7E, F**).

### Epithelial to Mesenchymal Transition, Cell Migration, Cell Signalling, and ECM Reorganization Genes Co-Correlates With Cav-1

To identify relevant genes correlating with Cav-1 in GB, we performed an analysis of the TCGA database using R2 (http://r2.amc.nl/). Specifically, from the advanced dataset selection panel of R2, we first selected the following database, TCGA-540-MAS5.0-u133a (4). We then identified the genes whose tumour expression correlated (either positively or negatively) with Cav-1 (False Discovery Rate correction at a p-value of < 0.01). We found 4879 genes correlating with Cav-1. Amongst these genes, 2194 were positively correlated, while 2686 were negatively correlated. Data for all these genes is available in Supplementary information (**Supplementary xlsx Excel Sheet**).

**Figure 8:**
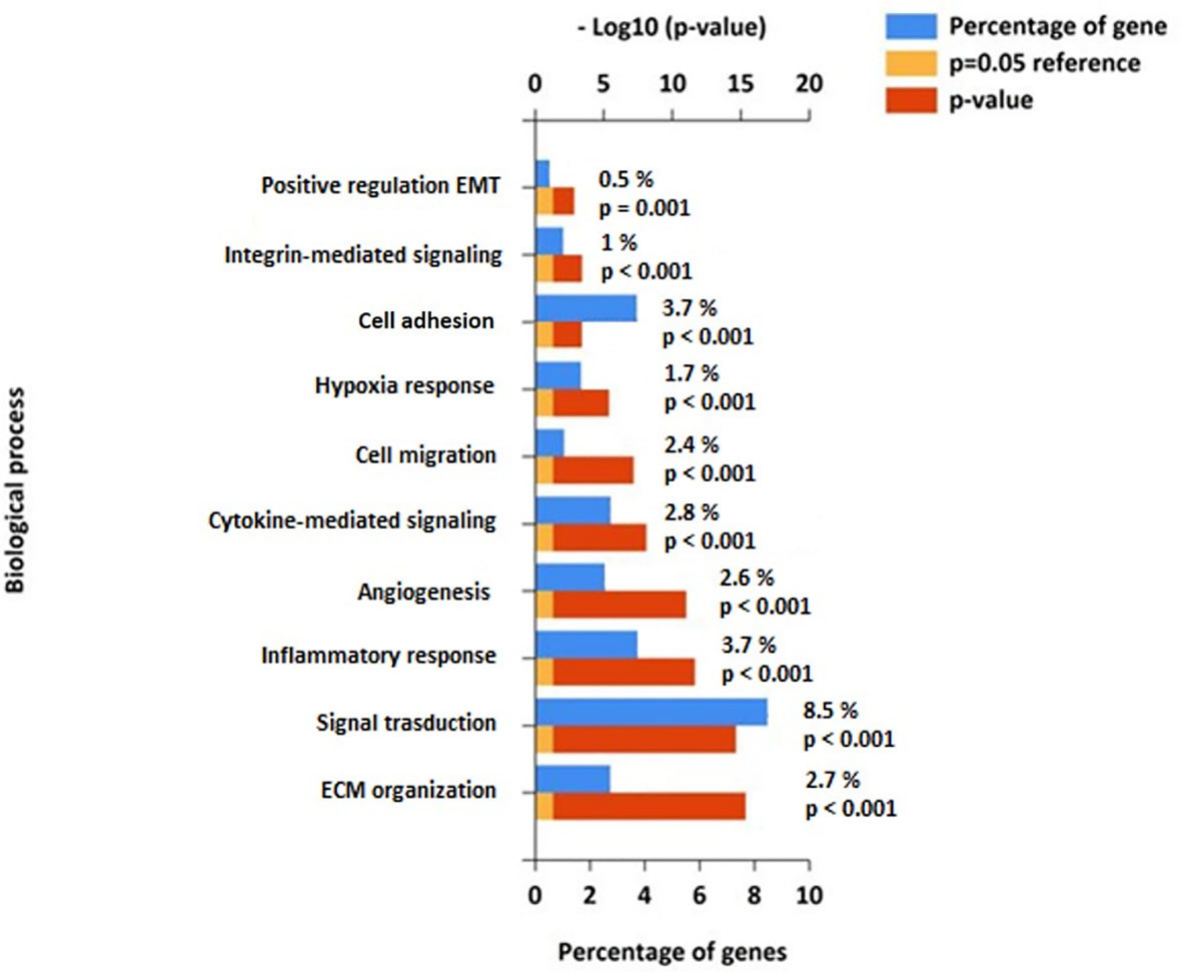
Functional pathway enrichment analysis of genes correlated to Cav-1 from the FunRich database (39). The Figure shows the top 10 biological highly populated canonical pathways from the analysis of 4879 genes related to Cav-1. Blue bars represent the percentage of genes assigned to the indicated pathway, orange bars show the reference p value (0.05), and red bars show the calculated p value of enrichment for the indicated term. Biological processes are ranked based on - Log10(p-value).

Given the involvement of Cav-1 in cellular signal transduction and cancer (15), we next undertook a pathway enrichment analysis of the above correlated findings using Fun Rich tools (39). This enrichment analysis of Cav-1 correlated DEGs highlighted an over-representation within 10 biological pathways shown in **Figure 8**. A substantial part of the correlated DEGs related to pathways for: Signal Transduction (8.5%), Cell Adhesion (3.7%), ECM organization (2.7%), Inflammatory response and Cytokine signalling (3.7% and 2.8%, respectively) and Cell migration (2.6%) circumstantially corroborating the role of Cav-1 in driving a range of different biological processes (**Figure 8**).

To address the putative interaction of Cav-1 with these molecules within the context of GB we next explored the relevance of certain biological signalling molecules identified above from the pathway enrichment (**Figure 8**). Specifically, we focused on those genes within the biological processes related to of cell adhesion, ECM organization and EMT pathways. From here we identified 27 genes shown in **Table 2**: the Table also highlighting the corresponding R-value (correlation in expression with Cav-1) and the associated statistical P-value (R-p). These genes ranged from those involved in: cell-cell adhesion or genes related to extracellular matrix (ECM) organization - CTSB, CTSD, CTSH, CTSK, CTSL, CTSS, MMP-1, MMP2, MMP3, MMP7, MMP9, MMP10, MMP14, UPA, UPAR, TSP1; integrin -mediated signalling - CD44, ITGA3, ITGA5, ITGAV, ITGB1, ITGB3, ITGB5; protease inhibitors - PAI1, TIMP1, TIMP3. As part of the analysis we also included the mesenchymal markers Vimentin and E-Cadherin as indicators of EMT (60).

### Cav-1 as a Key Driver of Survival: Analysis Using Single and Paired Markers Shows Genes Whose Expression Is Required for Cav-1 Increase

To test whether the genes identified as correlating with Cav-1 in **Table 2** provide a prognostic indicator of GB patient survival in their own right, we undertook first univariate survival analysis. Univariate single marker survival analysis of our TCGA cohort showed that patients having high expression of those 17 genes identified in **Table 2** (column 4 with a tick symbol), i.e. ITGA5, ITGB5, UPAR, CD44, MMP1, MMP2, MMP7, MMP9, MMP10, MT1MMP, CTSD, CTSB, CTSL, UPA, TIMP1, PA1 and TSP1, had a significantly worse prognosis (median survival time, hazard ratio and Cox p-value are reported in **Supplementary Table S1**). For the genes ECAD, CTSH, TIMP3, univariate single marker survival analysis showed high expression to be associated with favourable prognosis (**Supplementary Table S1**). Cox regression analysis was undertaken to determine the prognostic value of Cav-1 when in combination with genes listed in **Table 2**. The results of this analysis are fully reported in **Supplementary Table S1**. In particular, we found MMP2, MMP9, PAI1 when combined with Cav-1 to produce as significantly greater HR than when either of these two markers were used alone; suggesting co-operative synergistic mechanisms of action (**Table 3** and **Supplementary Figure S4** for survival analysis). Notably from our panel of markers in **Table 2**, we identified the genes ITGAV, CTSH and CTSK, while not predictors of survival in a univariate analysis, showed a significant a powerful HR only when in combination with Cav-1 expression (**Table 3** and **Supplementary Figure S4** for survival analysis curve).

**Table 3:** Hazard ratio for pathway molecules (MMP2, MMP9, PAI1) that when combined with Cav-1 produced a significantly greater HR than when either of these two markers were used alone, or molecules (CTSH, CTSK, ITGAV) solely when in combination with Cav-1 expression showed a significant and powerful HR.

	Univariate Cox Regression Model						Composite Cox Regression Model					
	Cut point	Median survival (Days)	95%CI	Log Rank p-value	Cox – HR	Coxp-value		Median survival (Days)	95% CI	Log Rank p-value	Cox – HR	Coxp-value
**ITGAV**	High	427	333-453	0.05			CAV^high^X^high^	138	87-342	2e-06	CAV^high^ = 3.155	1e-05
							CAV^high^X^low^	231	124-NA			
	Low	342	269-419		0.674	0.06	CAV^low^X^high^	532	427-737		ITGAV^high^ = 0.629	0.025
							CAV^low^X^low^	357	269-439			
**MMP2**	High	357	313-439	0.04	1.984	0.03	CAV^high^X^high^	138	83-342	3e-06	CAV^high^ = 3.456	4.7e-06
							CAV^high^X^low^	385	124-NA			
	Low	543	414-NA				CAV^low^X^high^	419	333-480		MMP2^high^ = 2.320	0.013
							CAV^low^X^low^	672	478-NA			
**MMP9**	High	359	313-439	0.04	1.789	0.03	CAV^high^X^high^	138	94-342	2e-05	CAV^high^ = 3.086	1.7e-05
							CAV^high^X^low^	148	87-NA			
	Low	737	357-NA				CAV^low^X^high^	414	333-480		MMP9^high^= 1.869	0.029
							CAV^low^X^low^	737	419-NA			
**CTSH**	High	543	269-NA	0.02			CAV^high^X^high^	233	62-NA	6e-06	CAV^high^ = 3.3311	5.7e-06
							CAV^high^X^low^	131	87-NA			
	Low	380	316-439		2.059	0.02	CAV^low^X^high^	570	505-NA		CTSH^high^ = 0.429	0.0097
							CAV^low^X^low^	414	342-478			
**CTSK**	High	323	270-439	0.09	1.418	0.09	CAV^high^X^high^	138	76-NA	1e-05	CAV^high^ = 3.096	1.8-e05
							CAV^high^X^low^	148	87-NA			
	Low	427	360-543				CAV^low^X^high^	414	313-480		CTSK^high^ = 1.484	0.06
							CAV^low^X^low^	448	399-772			
**PAI1**	High	231	146-485	0.03	1.715	0.04	CAV^high^X^high^	150	94-NA	2e-05	CAV^high^ = 3.215	0.00097
							CAV^high^X^low^	106	82-NA			
	Low	419	359-480				CAV^low^X^high^	313	164-NA		PAI1^high^ = 1.110	0.75732
							CAV^low^X^low^	427	360-489			

Median Survival and 95% Confidence Interval (CI), Log Rank test p value, Cox Hazard Ratio (HR) and corresponding p value are listed in the **Table 3**. Left panel: univariate analysis with genes shortlisted from **Table 2**. Right panel: composite multivariate regression analysis with Cav-1 expression. Bold values for Cox-HR and Cox p-value indicate where the composite analysis of X gene "high" combined with Cav-1 gene "high" results in a significantly greater HR than when either X gene or Cav-1 gene were used alone (note HR for Cav-1 alone < 2.99 from **Figure 2**).

## Discussion

Survival rates at 5-years for GB patients are reported at 3–5% with no notable improvement over the last three decades. Clinical-pathological parameters recently summarized by WHO to classify CNS tumours have been adopted in GB diagnosis (13), however further progress are needed to identify discriminative biomarkers for patient stratification both in terms of treatment options and prognostication.

Cav-1 is a regulator of multiple signal transduction events and cytoskeletal dynamics, and at least in preclinical models is reported to modulate several signalling pathways to promote and/or suppress the malignant phenotype. Two previous studies (see below) (34, 61) have reported Cav-1 in the context of the GB survival although Cox proportional hazard model methodology was not a feature of the work: Chen et al. (61) performing immunocytochemistry on tissues from 42 patients explored the relationship between of Cav-1 and HIF-1α. They showed expression levels of HIF-1α and Cav-1 to be upregulated in IDH-wild type tumours, and Cav-1 levels to be significantly correlated with high HIF-1α expression. Overexpression of HIF-1α and Cav-1 were each individually associated with a poor prognosis. Pu et al. (34) examined the expression of Cav-1 and Cavin-1 (a caveolae-related protein), and found both to be increased in GB with a higher expression of these caveola-forming proteins associated with shorter survival time. Both studies affirming Cav-1 as a potential biomarker for GB survival.

In this current work using clinical and genomic data information from the TCGA and CGGA databases of defined cohorts of primary GB patients, we show that Cav-1 expression positively correlates with shortened survival and importantly for the first time serves as a strong significant independent predictor of poor outcome (HR of 2.985 and 1.90, respectively). Intriguingly, despite well-established gender differences in the incidence of GB, with males showing the greater incidence (62), we report here that high Cav-1 tumour expression is associated with a much greater risk to females, approximate 70% reduction in median survival. Recent insights using large-scale analyses of TCGA data have revealed gender differences in the GB tumour at the molecular level and in response to therapy (63). In this context we note Cav-1 has been found to respond to oestrogenic factors (64). Apart from the gender-specific tumour types, this finding reinforces that subtle gender differences are often obscured in large-scale analysis (63) and should receive greater attention when stratifying data.

In exploring the interrelationship of Cav-1 with clinical prognostic indicators our findings showed a high expression of Cav-1 associated with high grade histological type and not surprisingly high KPS patient score. We further found Cav-1 association with IDH wild type status. With respect to GB molecular subtype classification (5), some 85 patients of the original 152 cohort were sub-typed, and within which we found Cav-1 tumour expression to be significantly greater in the mesenchymal and proneural GB subtypes (combined 60% of the population). Recent gene expression data has shown Cav-1 to be one of the top genes upregulated in the invasive GB phenotype and especially so in tumours with a marked mesenchymal signature (65). Our own data showed high Cav-1 expression to be associated with a significantly poor survival outcome in mesenchymal (HR 3.06) and proneural (HR 2.65) subtypes but not in the classical or neural subtypes; Pu et al. (34) similarly showed increased Cav-1 expression in mesenchymal GB. The GB subtypes, despite sharing the same essential characteristics, do show distinct phenotypic and genotypic differences, with the mesenchymal subtype correlating with poor outcome and resistance to irradiation (66). The strength of Cav-1 as a prognostic indicator in the mesenchymal GB cohort suggests it may have a profound role in inducing and/or maintaining the mesenchymal GB signature.

We went on to explore the risk stratification of patient tumours expressing high levels of Cav-1. Initially against clinical parameters such as IDH1 status, PTEN, EGFR-vIII, MGMT, TP53, all established markers for diagnosis, prognosis and response to therapy in GB molecular subgroup (13). The multivariate model revealed strong associations of Cav-1 with IDH1-wt, PTEN-mut and EGFR-vIII-amplified, with composite covariate analysis showing high Cav-1 expression combined with either of the above markers to result in a statistically shorter patient survival than when any of the markers were used alone (composite HR of 11.48, 4.77, 3.53, respectively). This is consistent with Cav-1 involvement in multiple independent cellular pathways. In melanoma, for example, the genetic mutation of PTEN increases Cav-1-mediated dissociation of β-catenin from membranous E-cadherin bypassing senescence process and promoting metastasis (67). Contributing to an aggressive phenotype the most common EGFR variant in GB, EGFR-vIII, is characterised by a deletion in the extracellular domain leading to the expression of a constitutively autophosphorylated receptor unable to bind ligand. Glioblastoma cells have been reported to have high Cav-1 expression in association with EGFR-vIII (mut) (68). However, while wild-type EGFR colocalises with Cav-1 membrane domains in a phosphorylation-dependent manner with functional consequences (56–58), EGFR-vIII appears to be predominantly cytoplasmic and not associated with the Cav-1 membrane domains; how high Cav-1 results in functional synergy with EGFR-vIII remains to be determined.

Hypoxia is commonly found in the tumour microenvironment and represents a critical feature in cancer progression (69). In high grade glioma, hypoxic and necrotic areas are typically surrounded by hypercellular regions of ‘pseudopalisading’ waves of tumour cells migrating away from the hypoxic tumour mass and infiltrating normal brain tissue (41, 43, 70); hypoxia and the ‘pseudopalisading’ morphology is associated with poor prognosis and resistance to therapies (71). We report here the higher expression of Cav-1 within the peri-necrotic and pseudo-palisading areas of the GB tumour, areas also expressing high levels of HIF-1α factor. Bourseau-Guilmain et al. (72) initially described the expression of Cav-1 in hypoxic region of GB tumours. Confirmed by Chen W et al. (61) where the overexpression of both HIF-1α and Cav-1 in the IDH-wild type GB patients was associated with shorter patient survival. Hypoxia regulates membrane protein endocytosis through a Cav-1-mediated process in some cancer cell lines (70, 72). More relevant, Cav-1 is directly activated by hypoxia-inducible factor (HIF) (73) with hypoxia-dependent migration tumour cell lines blocked upon Cav-1 knock-down and some evidence that hypoxia-induced migration and invasion of metastatic cancer cells at least, require HIF1α-dependent induction of Cav-1 expression and src family kinase activation (20). We also report higher expression of Cav-1 within endothelial cells of the hyperplastic vessels around necrosis of the tumour; the significance of Cav-1 with the tumour vasculature explored in other cancers (65–68) and with some evidence (74) that Cav-1 can regulate endothelial cell plasticity, however, the functional implication of the up- or downregulation of Cav-1 in angiogenesis and associated tumour growth requires further work in a tumour-specific manner.

To explore gene co-operativity between Cav-1 in driving poor outcome in GB patients, we undertook a correlation analysis (either positive or negative) with Cav-1 and found 4879 differentially expressed genes (DEGs) which significantly correlate with Cav-1 expression. As expected, when inferred for pathway analysis, these genes revealed a substantial enrichment for pathways involved in Signal Transduction (8.5%), Cell Adhesion (3.7%), ECM Organisation (2.7%), Inflammatory Response (3.7%), Cytokine Signalling (2.8%) and Cell Migration (2.6%); a not unsurprising analysis given the role of Cav-1 in driving a range of different biological processes associated with cancer (75, 76) including EMT processes (77). Although the specific mechanism by which Cav-1 facilitates GB progression is still unclear, the pathway analysis demonstrates strong association between Cav-1 and genes whose enrichment signature corresponds to cell adhesion and ECM organisation, with Cav-1 potentially serving as a “gatekeeper” to the triggering of downstream molecules for EMT progression (78, 79). Indeed, the *in-vitro* data we presented shows significantly decreased cell proliferation and notably decreased cell migration when Cav-1 was depleted by CRISP-Cas-9.

To date the significance of Cav-1 in prognostication when combined with other biomarkers has received little attention. The EMT phenomenon is an important feature in glioma progression and survival (80), adopting a data-driven inductive approach building on the above pathway analysis we explored the combination of Cav-1 with genes associated with adhesion, ECM organisation and EMT pathways. We found 27 main representative genes (**Table 2**) of the pathways above that showed a strong correlation in GB with Cav-1; importantly for 17 of these genes (i.e. ITGA5, ITGB5, UPAR, CD44, MMP1, MMP2, MMP7, MMP9, MMP10, MT1MMP, CTSD, CTSB, CSTL, UPA, TIMP1, PA1 and TSP1) their expression as a single marker was associated with a significantly worse prognosis (Univariate survival analysis), whereas three genes (ECAD,CTSH and TIMP3) whose single expression was instead associated with a more favourable prognosis. The aforementioned 17 genes have been linked in various experimental settings to be involved in gliomagenesis and aggressiveness (28, 70–74, 81–83). However, we then advanced our clinical understanding by undertaking cox regression (Hazard model) analysis to determine the prognostic value of pairing of each of these genes with Cav-1 and how in various combinations this may serve GB patient prognostic stratification. Based on Hazard model, we identified a strong synergy of Cav-1 with each of the following three genes, MMP2 (combined high expression HR 3.456), MMP9 (combined high expression HR 3.086) and PAI1 (combined high expression 3.215). Each of the MMP2 (84), MMP9 (85) and PAI1 (86) have previously been proposed as potential biomarkers related to glioma survival. Our result not only confirm the prognostication role of these molecules as independent markers but importantly provide new insight of their functional combination with the high expression of Cav-1, leading to a significantly greater HR than when either of these two markers are used alone. Cav-1 expression has previously been reported to lead to activation of MMP2 and MMP9 promoting invasion in hepatocellular carcinoma cell lines (87) and non-small lung carcinoma (88). Intriguingly, at least in the glioma U87 cell line (27), PAI1 has been shown to be mechanistically regulated in an inverse manner by Cav-1, whereas in prostate cancer PAI1 has been shown to have a positive correlation with Cav-1 (89) and within which the mesenchymal signature phenotype included high expression of Cav-1 and markers of EMT transition inc. PAI1 and integrins.

Cathepsins in cancer cells have been found to be functionally associated with binding partners within caveolae during the processes of lysosomal/endosomal cycling (90) and during the promotion of cell migration (56). In particular, CTSK gene has been found upregulated in GB cell lines and GB tissue samples although expression has not been used for survival prognostication. Here we found high CTSK gene expression while not a predictor of poor survival in a single analysis (**Table 3**) appeared to influence the Cav-1 when the two markers combined. Specifically, high CTSK in combination with high Cav-1 resulted in a significantly greater HR (3.096) (**Table 3**) than the HR value of Cav-1 alone. In contrast, low CTSH gene expression appeared to be a predictor of poor survival in a single analysis and when in combination with high Cav-1 resulted in a significantly greater HR (3.331) (**Table 3**). Cav-1 has a structural role within membranes acting act as a link between a variety of cell-surface receptors lacking a cytoplasmic domain to intracellular signalling pathways such as integrins (91). ITGAV activation, which has been shown to act either as tumour suppressor or oncogéne (92). Our hazard model showed an interaction between Cav-1 and ITGAV: we found low ITGAV gene expression, while not a predictor of poor survival, although tending toward this in a single analysis (**Table 3**). However, similar to CTSH when low ITGAV was combined with high Cav-1 a significantly greater HR (3.155) resulted.

In summary, the current work confirms high expression of Cav-1 in the GB tumour to be a significant independent predictor of shortened survival in TCGA and CGGA database. Cav-1 staining in GB tumours shows a strong cytoplasmatic/membranous positivity within tumour cells and associated endothelium. High expression for Cav-1 is particularly noted within hyperplastic blood vessels and microvasculature proliferations as well as within the tumours’ peri-necrotic and pseudo-palisading zones. We found female patients expressing high tumour levels of Cav-1 displayed a significantly shorter median survival time compared to male patients expressing high levels of Cav-1 (median survival 90.5 days *vs* 320 days: HR= 3.145). Further, the negative effect upon survival of high Cav-1 levels in the GB tumour was most evident in the Proneural and Mesenchymal GB subtypes. Increased tumour expression of Cav-1 was also associated with the IDH-wild type patient cohort with the composite co-variate of high Cav-1 expression and IDH-wild type status producing an extraordinarily powerful and probability of poor outcome (HR = 11.4). Moreover, Cav-1 appears to be linked to many signalling entities within the GB tumour and as such this work begins to substantiate Cav-1 or its associated signalling partners as candidate target for GB new drug discovery which remains an unmet medical need.

## Data Availability Statement

The datasets presented in this study can be found in online repositories. The names of the repository/repositories and accession number(s) can be found in the article/**Supplementary Material**.

## Author Contributions

Conceptualization, MG. Methodology, MG, PC, CM, and CN. Formal analysis, PC and CM. Investigation, CM, PC and CN. Data curation, CM, PC. Writing—original draft preparation, CM and PC. Writing—review and editing, PC, MG and GJP. Funding acquisition, MG and GJP. All authors contributed to the article and approved the submitted version.

## Funding

This research was funded by Cancer Research Wales (Ed Evans Brain Tumour Scholarship) Brain Tumour Research and the Jake McCarthy Foundation.

## Conflict of Interest

The authors declare that the research was conducted in the absence of any commercial or financial relationships that could be construed as a potential conflict of interest.

## Publisher’s Note

All claims expressed in this article are solely those of the authors and do not necessarily represent those of their affiliated organizations, or those of the publisher, the editors and the reviewers. Any product that may be evaluated in this article, or claim that may be made by its manufacturer, is not guaranteed or endorsed by the publisher.

## Supplementary Material

Supplementary Figure 1 Overall survival in 155 GB patients by gender and tumour Cav-1 expression status. **S1(A)**. Overall survival curves for GB patients by gender. The univariate analysis revealed no difference in survival by gender (median survival: male 378 days *vs* female 399 days; **S1(B)**. Survival combining both tumour Cav-1 expression levels (high, Cav+; low, Cav-) and gender. Multivariate analysis showed female patients expressing high tumour levels of Cav-1 to display a significantly shorter median survival time compared to male patients expressing high levels of Cav-1 (median survival 90.5 days *vs* 320 days: HR 3.145, P=0.0000015). No gender-based adverse outcome on survival was seen in patients whose tumours expressed low levels of Cav-1, i.e. median survival (M) 427 days *vs* (F) 419 days (HR 1.471, P=0.066). Vertical lines connect median survival times. The number of patients alive at each time-point is reported under the plots. Tables show Log Rank p-value for the evaluation of the curves statistical difference; median survival for each group is reported together with the confidence interval (CI), whereas Cox hazard ratio for the referring group is coupled with its p-value. Plot and analysis were achieved through Survimor R package

Supplementary Figure 2 **(A–D)** From the TCGA dataset of GB patients Cav-1 expression in patients’ GB tumours were correlated with respect to the sub-groups for **(A)** EGFR-vIII, **(B)** PTEN, **(C)** MGMT and **(D)** TP53 molecular status. Boxes represent median (horizontal line) Cav-1 tumour expression (expressed as log2-fold) and 25th and 75th percentiles (error bars). Data were analysed using Student T-test (unpaired) for comparison of two groups.

Supplementary Figure 3 In-situ hybridization (ISH) for Cav-1 in mesenchymal and proneural patients’ sections retrieved from IVY database **(A, B)**. Representative ISH of Cav-1 expression shows specific pattern of expression for features isolated by LMD and subsequently assessed by RNA-seq approach. ML annotations for ISH and H&E (haematoxylin and eosin stain), and H&E adjacent to ISH. Colour code: blue, LE; purple, IT; green, CT; light blue, PN; turquoise, PAN; orange, HBV; red/magenta, MpVs; black, necrosis. HBVs, Hyperplastic blood vessels in cellular tumour; MpVs, Microvascular proliferation; PN, Pseudo-palisading cells around necrosis; IT, Infiltrating tumour; LE, Leading edge; CT, Cellular tumour.

Supplementary Figure 4 **(A–N)**. Kaplan Meier plot of ITGAV, CTSH, CTSK, MMP2, MMP9, PAI1 upon GB patient survival and their correlation with Cav-1. Left: for each gene, Kaplan Meier plot of Overall survival of GB patients expressing high and low levels of the selected genes. Vertical lines connect median survival times. The number of patients alive at each time-point is reported under the plots. Tables show Log Rank p-value for the evaluation of the curves statistical difference; median survival for each group is reported together with the confidence interval (CI), whereas Cox hazard ratio for the referring group is coupled with its p-value. Plot and analysis were achieved through Survimer R package.

Supplementary Table 1 Univariate and multivariate analysis on 27 genes combined with Cav-1 expression. Median Survival and 95% Confidence Interval (CI), Log Rank test p value, Cox Hazard Ratio (HR) and corresponding p-value are listed in the table. Left panel: univariate analysis with 27 gene shortlisted from **Table 2** (main Manuscript). Right panel: combined multivariate regression analysis with Cav-1 expression.

Supplementary Table 2 List of genes correlating (positivelly and negativelly) with Cav-1.
