# Supplementary figures and images for: Caveolin-1, a Key Mediator Across Multiple Pathways in Glioblastoma and an Independent Negative Biomarker of Patient Survival
Source: Front Oncol. 2021 Aug 20;11:701933. doi: 10.3389/fonc.2021.701933 (PMC8417742; doi:10.3389/fonc.2021.701933)

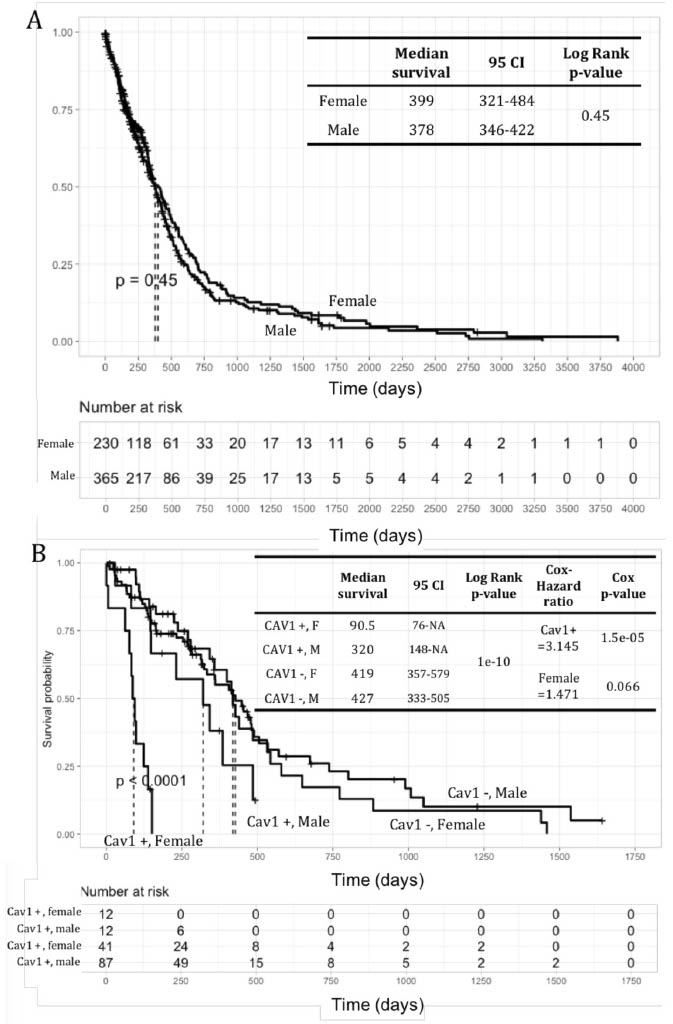

Supplement: Supplementary Figure 1 — Overall survival in 155 GB patients by gender and tumour Cav-1 expression status. S1(A). Overall survival curves for GB patients by gender. The univariate analysis revealed no difference in survival by gender (median survival: male 378 days vs female 399 days; S1(B). Survival combining both tumour Cav-1 expression levels (high, Cav+; low, Cav-) and gender. Multivariate analysis showed female patients expressing high tumour levels of Cav-1 to display a significantly shorter median survival time compared to male patients expressing high levels of Cav-1 (median survival 90.5 days vs 320 days: HR 3.145, P=0.0000015). No gender-based adverse outcome on survival was seen in patients whose tumours expressed low levels of Cav-1, i.e. median survival (M) 427 days vs (F) 419 days (HR 1.471, P=0.066). Vertical lines connect median survival times. The number of patients alive at each time-point is reported under the plots. Tables show Log Rank p-value for the evaluation of the curves statistical difference; median survival for each group is reported together with the confidence interval (CI), whereas Cox hazard ratio for the referring group is coupled with its p-value. Plot and analysis were achieved through Survimor R package [file Image_1.jpg]

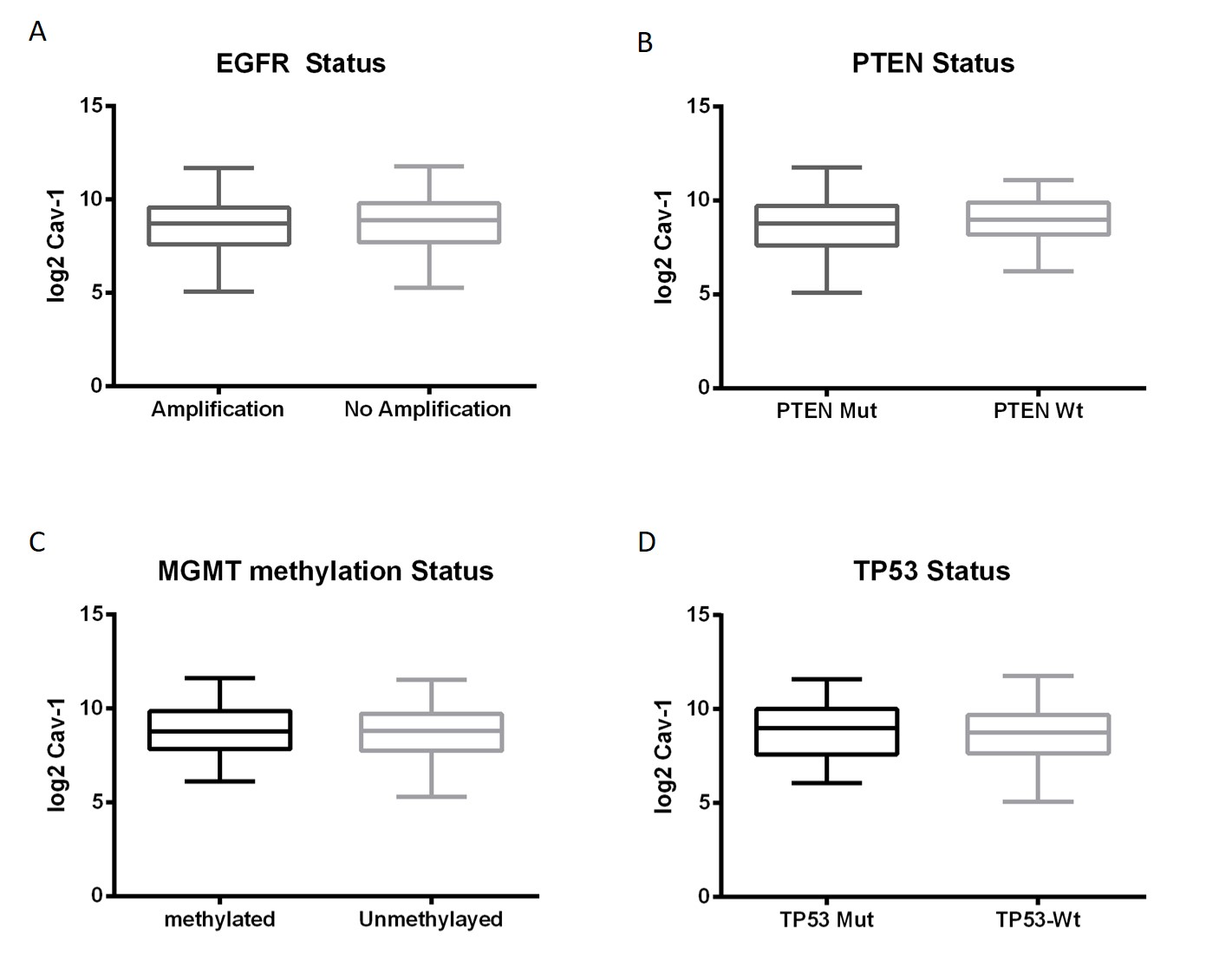

Supplement: Supplementary Figure 2 — (A–D) From the TCGA dataset of GB patients Cav-1 expression in patients’ GB tumours were correlated with respect to the sub-groups for (A) EGFR-vIII, (B) PTEN, (C) MGMT and (D) TP53 molecular status. Boxes represent median (horizontal line) Cav-1 tumour expression (expressed as log2-fold) and 25th and 75th percentiles (error bars). Data were analysed using Student T-test (unpaired) for comparison of two groups. [file Image_2.jpeg]

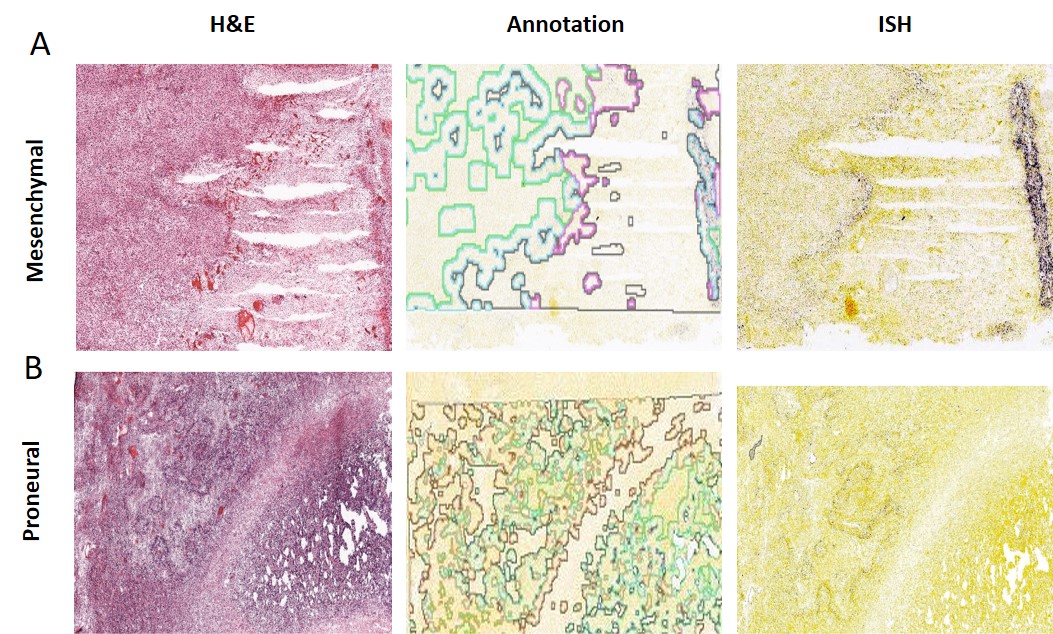

Supplement: Supplementary Figure 3 — In-situ hybridization (ISH) for Cav-1 in mesenchymal and proneural patients’ sections retrieved from IVY database (A, B). Representative ISH of Cav-1 expression shows specific pattern of expression for features isolated by LMD and subsequently assessed by RNA-seq approach. ML annotations for ISH and H&E (haematoxylin and eosin stain), and H&E adjacent to ISH. Colour code: blue, LE; purple, IT; green, CT; light blue, PN; turquoise, PAN; orange, HBV; red/magenta, MpVs; black, necrosis. HBVs, Hyperplastic blood vessels in cellular tumour; MpVs, Microvascular proliferation; PN, Pseudo-palisading cells around necrosis; IT, Infiltrating tumour; LE, Leading edge; CT, Cellular tumour. [file Image_3.jpeg]

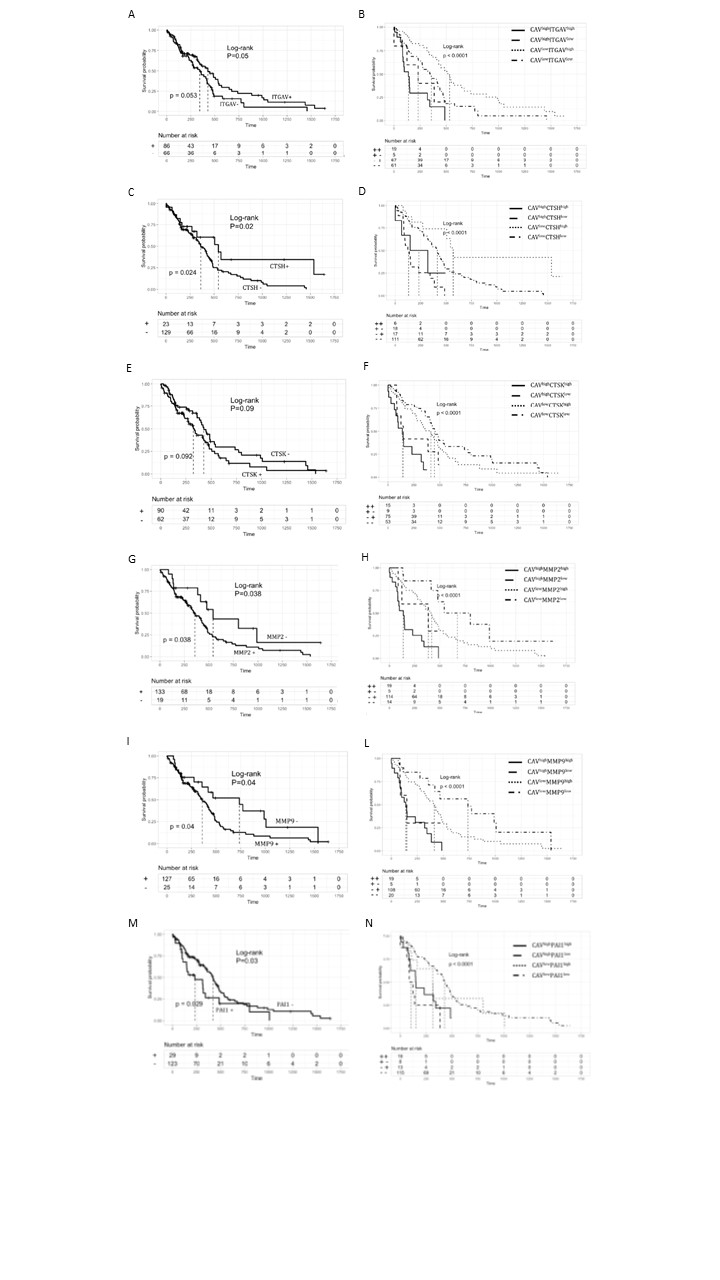

Supplement: Supplementary Figure 4 — (A–N). Kaplan Meier plot of ITGAV, CTSH, CTSK, MMP2, MMP9, PAI1 upon GB patient survival and their correlation with Cav-1. Left: for each gene, Kaplan Meier plot of Overall survival of GB patients expressing high and low levels of the selected genes. Vertical lines connect median survival times. The number of patients alive at each time-point is reported under the plots. Tables show Log Rank p-value for the evaluation of the curves statistical difference; median survival for each group is reported together with the confidence interval (CI), whereas Cox hazard ratio for the referring group is coupled with its p-value. Plot and analysis were achieved through Survimer R package. [file Image_4.jpeg]
