# Supplementary material for: Caveolin-1, a Key Mediator Across Multiple Pathways in Glioblastoma and an Independent Negative Biomarker of Patient Survival
Source: Front Oncol. 2021 Aug 20;11:701933. doi: 10.3389/fonc.2021.701933 (PMC8417742; doi:10.3389/fonc.2021.701933)
Supplement: Supplementary Table 1 — Univariate and multivariate analysis on 27 genes combined with Cav-1 expression. Median Survival and 95% Confidence Interval (CI), Log Rank test p value, Cox Hazard Ratio (HR) and corresponding p-value are listed in the table. Left panel: univariate analysis with 27 gene shortlisted from Table 2 (main Manuscript). Right panel: combined multivariate regression analysis with Cav-1 expression. [file Table_1.docx]

**Supplementary Table S1.**

|  | **Univariate cox regression** | | | | | | **Composite cox regression** | | | | | |
| --- | --- | --- | --- | --- | --- | --- | --- | --- | --- | --- | --- | --- |
|  | **Cut point** | **Median survival** | **95 CI** | **Log Rank**  **p-value** | **Cox- HR** | **Cox**  **p-value** |  | **Median survival** | **95 CI** | **Log Rank**  **p-value** | **Cox- HR** | **Cox**  **p-value** |
| **ITGAV** | High | 427 | 333-543 | 0.05 |  |  | CAV^high^X^high^ | 138 | 87-342 | 2e-06 | Cav ^High^  =  3.155 | 1e -05 |
|  |  |  |  |  |  |  | CAV^high^X^low^ | 231 | 124-NA |  |  |  |
|  | Low | 342 | 269-419 |  | 0.674 | 0.06 | CAV^low^X^high^ | 532 | 427-737 |  | ITGAV ^High^  =  0.629 | 0.025 |
|  |  |  |  |  |  |  | CAV^low^X^low^ | 357 | 269-439 |  |  |  |
| **ITGA3** | High | 360 | 279-454 | 0.07 | 1.527 | 0.06 | CAV^high^X^high^ | 142 | 94-342 | 4e-05 | Cav ^high^  =  2.591 | 0.0002 |
|  |  |  |  |  |  |  | CAV^high^X^low^ | - | - |  |  |  |
|  | Low | 427 | 333-772 |  |  |  | CAV^low^X^high^ | 419 | 342-489 |  | ITGA3^high^  =  1.497 | 0.3 |
|  |  |  |  |  |  |  | CAV^low^X^low^ | 427 | 333-772 |  |  |  |
| **ITGA5** | High | 316 | 231-454 | 0.007 | 1.757 | 0.008 | CAV^high^X^high^ | 148 | 124-NA | 4e-04 | Cav ^high^  =  2.591 | 0.00045 |
|  |  |  |  |  |  |  | CAV^high^X^low^ | 87 | 62-NA |  |  |  |
|  | Low | 419 | 359-532 |  |  |  | CAV^low^X^high^ | 406 | 280-489 |  | ITGA5^high^  =  1.497 | 0.067 |
|  |  |  |  |  |  |  | CAV^low^X^low^ | 427 | 360-674 |  |  |  |
| **ITGB1** | High | 342 | 275-478 | 0.2 | 1.344 | 0.2 | CAV^high^X^high^ | 146 | 98-385 | 6e-06 | Cav ^high^  =  2.865 | 0.0001 |
|  |  |  |  |  |  |  | CAV^high^X^low^ | 84 | 82-NA |  |  |  |
|  | Low | 419 | 359-648 |  |  |  | CAV^low^X^high^ | 406 | 316-505 |  | ITGB1^high^  =  1.135 | 0.583 |
|  |  |  |  |  |  |  | CAV^low^X^low^ | 427 | 360-737 |  |  |  |
| **ITGB3** | High | 485 | 313-737 | 0.07 |  |  | CAV^high^X^high^ | 146 | 94-385 | 3e-05 | Cav ^high^  =  2.710 | 0.00017 |
|  |  |  |  |  |  |  | CAV^high^X^low^ | 87 | 62-NA |  |  |  |
|  | Low | 359 | 279-427 |  | 1.460 | 0.07 | CAV^low^X^high^ | 427 | 313-480 |  | ITGB3^high^  =  1.531 | 0.09 |
|  |  |  |  |  |  |  | CAV^low^X^low^ | 505 | 360-1050 |  |  |  |
| **ITGB5** | High | 316 | 231-478 | 0.02 | 1.715 | 0.02 | CAV^high^X^high^ | 138 | 94-NA | 3e-05 | Cav ^high^  =  2.611 | 0.00043 |
|  |  |  |  |  |  |  | CAV^high^X^low^ | 148 | 83-NA |  |  |  |
|  | Low | 439 | 414-772 |  |  |  | CAV^low^X^high^ | 399 | 280-485 |  | ITGB5^high^  =  1.420 | 0.112 |
|  |  |  |  |  |  |  | CAV^low^X^low^ | 468 | 406-648 |  |  |  |
| **UPAR** | High | 316 | 237-399 | 1e-04 | 2.457 | 2e-04 | CAV^high^X^high^ | 146 | 96-342 | 4e-07 | Cav ^high^  =  2.294 | 0.0021 |
|  |  |  |  |  |  |  | CAV^high^X^low^ | 82 | NA-NA |  |  |  |
|  | Low | 480 | 427-1008 |  |  |  | CAV^low^X^high^ | 342 | 279-448 |  | uPAR ^high^  =  2.123 | 0.0026 |
|  |  |  |  |  |  |  | CAV^low^X^low^ | 480 | 427-1008 |  |  |  |
| **CD44** | High | 320 | 237-478 | 0.04 | 1.536 | 0.04 | CAV^high^X^high^ | 142 | 94-342 | 5e-05 | CAV ^high^  =  2.688 | 0.0005 |
|  |  |  |  |  |  |  | CAV^high^X^low^ | - | - |  |  |  |
|  | Low | 419 | 359-570 |  |  |  | CAV^low^X^high^ | 427 | 313-535 |  | CD44^high^  =  1.221 | 0.3779 |
|  |  |  |  |  |  |  | CAV^low^X^low^ | 419 | 359-570 |  |  |  |
| **ECAD** | High | 441 | 342-478 | 0.009 |  |  | CAV^high^X^high^ | 135 | 87-NA | 7e-05 | Cav ^high^  =  2.674 | 0.0004 |
|  |  |  |  |  |  |  | CAV^high^X^low^ | 184 | 76-NA |  |  |  |
|  | Low | 231 | 138-NA |  | 0.411 | 0.02 | CAV^low^X^high^ | 427 | 360-489 |  | ECAD ^high^  =  0.605 | 0.1736 |
|  |  |  |  |  |  |  | CAV^low^X^low^ | 316 | 138-NA |  |  |  |
| **MMP1** | High | 313 | 231-480 | 0.01 | 1.672 | 0.02 | CAV^high^X^high^ | 150 | 138-NA | 3e-05 | Cav ^high^  =  2.667 | 0.00027 |
|  |  |  |  |  |  |  | CAV^high^X^low^ | 85 | 62-NA |  |  |  |
|  | Low | 419 | 360-532 |  |  |  | CAV^low^X^high^ | 359 | 270-505 |  | MMP1 ^high^  =  1.445 | 0.09427 |
|  |  |  |  |  |  |  | CAV^low^X^low^ | 427 | 399-648 |  |  |  |
| **MMP2** | High | 357 | 313-439 | 0.04 | 1.984 | 0.03 | CAV^high^X^high^ | 138 | 83-342 | 3e-06 | Cav ^high^  =  3.456 | 4.7e-06 |
|  |  |  |  |  |  |  | CAV^high^X^low^ | 385 | 124-NA |  |  |  |
|  | Low | 543 | 414-NA |  |  |  | CAV^low^X^high^ | 419 | 333-480 |  | MMP2 ^high^  =  2.320 | 0.013 |
|  |  |  |  |  |  |  | CAV^low^X^low^ | 672 | 478-NA |  |  |  |
| **MMP3** | High | 485 | 313-737 | 0.07 |  |  | CAV^high^X^high^ | 146 | 98-NA | 1e-05 | Cav ^high^  =  2.625 | 6.5e-06 |
|  |  |  |  |  |  |  | CAV^high^X^low^ | 94 | 83-NA |  |  |  |
|  | Low | 359 | 279-427 |  | 0.685 | 0.07 | CAV^low^X^high^ | 543 | 342-883 |  | MMP3^high^  =  1.641 | 0.019 |
|  |  |  |  |  |  |  | CAV^low^X^low^ | 406 | 333-448 |  |  |  |
| **MMP7** | High | 316 | 237-439 | 0.02 | 1.645 | 0.02 | CAV^high^X^high^ | 146 | 94-342 | 1e-05 | Cav ^high^  =  2.625 | 0.00044 |
|  |  |  |  |  |  |  | CAV^high^X^low^ | 82 | NA-NA |  |  |  |
|  | Low | 468 | 414-772 |  |  |  | CAV^low^X^high^ | 359 | 275-505 |  | MMP7^high^  =  1.368 | 0.17435 |
|  |  |  |  |  |  |  | CAV^low^X^low^ | 468 | 414-772 |  |  |  |
| **MMP9** | High | 359 | 313-439 | 0.04 | 1.789 | 0.03 | CAV^high^X^high^ | 138 | 94-342 | 2e-05 | Cav ^high^  =  3.086 | 1.7e-05 |
|  |  |  |  |  |  |  | CAV^high^X^low^ | 148 | 87-NA |  |  |  |
|  | Low | 737 | 357-NA |  |  |  | CAV^low^X^high^ | 414 | 333-480 |  | MMP9 ^high^  =  1.869 | 0.029 |
|  |  |  |  |  |  |  | CAV^low^X^low^ | 737 | 419-NA |  |  |  |
| **MMP10** | High | 320 | 237-478 | 0.02 | 1.653 | 0.02 | CAV^high^X^high^ | 142 | 94-342 | 7e-05 | Cav ^high^  =  2.703 | 0.00019 |
|  |  |  |  |  |  |  | CAV^high^X^low^ | 236 | 87-NA |  |  |  |
|  | Low | 427 | 399-648 |  |  |  | CAV^low^X^high^ | 406 | 280-505 |  | MMMP10 ^high^  =  1.456 | 0.10277 |
|  |  |  |  |  |  |  | CAV^low^X^low^ | 427 | 414-772 |  |  |  |
| **MT1MMP** | High | 359 | 316-439 | 0.02 | 2.451 | 0.008 | CAV^high^X^high^ | 142 | 94-342 | 9e-06 | Cav ^high^  =  2.762 | 0.00011 |
|  |  |  |  |  |  |  | CAV^high^X^low^ | - | - |  |  |  |
|  | Low | 772 | 414-NA |  |  |  | CAV^low^X^high^ | 419 | 342-478 |  | MT1MMP ^High^  =  2.212 | 0.0378 |
|  |  |  |  |  |  |  | CAV^low^X^low^ | 772 | 414-NA |  |  |  |
| **CTSB** | High | 333 | 270-427 | 0.01 | 2.155 | 0.017 | CAV^high^X^high^ | 142 | 94-342 | 1e-05 | Cav ^high^  =  2.681 | 0.0002 |
|  |  |  |  |  |  |  | CAV^high^X^low^ | - | - |  |  |  |
|  | Low | 478 | 454-NA |  |  |  | CAV^low^X^high^ | 399 | 316-485 |  | CTSB ^high^  =  1.883 | 0.053 |
|  |  |  |  |  |  |  | CAV^low^X^low^ | 478 | 454-NA |  |  |  |
| **CTSD** | High | 269 | 151-342 | 0.003 | 1.848 | 0.004 | CAV^high^X^high^ | 151 | 87-NA | 7e-07 | Cav ^high^  =  2.451 | 0.0015 |
|  |  |  |  |  |  |  | CAV^high^X^low^ | 124 | 82-NA |  |  |  |
|  | Low | 427 | 385-489 |  |  |  | CAV^low^X^high^ | 270 | 164-570 |  | CSTD ^high^  =  1.486 | 0.0807 |
|  |  |  |  |  |  |  | CAV^low^X^low^ | 454 | 419-532 |  |  |  |
| **CTSH** | High | 543 | 269-NA | 0.02 |  |  | CAV^high^X^high^ | 233 | 62-NA | 6e-06 | Cav ^high^  =  3.3311 | 5.7e-06 |
|  |  |  |  |  |  |  | CAV^high^X^low^ | 131 | 87-NA |  |  |  |
|  | Low | 360 | 316-439 |  | 2.059 | 0.02 | CAV^low^X^high^ | 570 | 505-NA |  | CTSH ^high^  =  0.429 | 0.0097 |
|  |  |  |  |  |  |  | CAV^low^X^low^ | 414 | 342-478 |  |  |  |
| **CTSK** | High | 323 | 270-439 | 0.09 | 1.418 | 0.09 | CAV^high^X^high^ | 138 | 76-NA | 1e-05 | Cav ^high^  =  3.096 | 1.8-e05 |
|  |  |  |  |  |  |  | CAV^high^X^low^ | 148 | 87-NA |  |  |  |
|  | Low | 427 | 360-543 |  |  |  | CAV^low^X^high^ | 414 | 313-480 |  | CTSK ^high^  =  1.484 | 0.06 |
|  |  |  |  |  |  |  | CAV^low^X^low^ | 448 | 399-772 |  |  |  |
| **CTSL** | High | 316 | 231-478 | 0.02 | 1.715 | 0.02 | CAV^high^X^high^ | 142 | 94-342 | 3e-05 | Cav ^high^  =  2.632 | 0.00036 |
|  |  |  |  |  |  |  | CAV^high^X^low^ | - | - |  |  |  |
|  | Low | 439 | 414-772 |  |  |  | CAV^low^X^high^ | 359 | 270-532 |  | CTSL^high^  =  1.443 | 0.136 |
|  |  |  |  |  |  |  | CAV^low^X^low^ | 439 | 414-772 |  |  |  |
| **CTSS** | High | 342 | 269-427 | 0.07 | 1.506 | 0.2 | CAV^high^X^high^ | 146 | 87-485 | 2e-05 | Cav ^high^  =  2.762 | 0.00015 |
|  |  |  |  |  |  |  | CAV^high^X^low^ | 124 | 82-NA |  |  |  |
|  | Low | 468 | 414-772 |  |  |  | CAV^low^X^high^ | 360 | 313-505 |  | CTSS ^high^  =  1.300 | 0.260 |
|  |  |  |  |  |  |  | CAV^low^X^low^ | 478 | 419-1050 |  |  |  |
| **UPA** | High | 320 | 237-448 | 0.03 | 1.580 | 0.03 | CAV^high^X^high^ | 146 | 94-385 | 2e-05 | Cav ^high^  =  2.667 | 0.00035 |
|  |  |  |  |  |  |  | CAV^high^X^low^ | 106 | 87-NA |  |  |  |
|  | Low | 427 | 357-737 |  |  |  | CAV^low^X^high^ | 419 | 280-489 |  | UPA^high^  =  1.314 | 0.227 |
|  |  |  |  |  |  |  | CAV^low^X^low^ | 427 | 360-737 |  |  |  |
| **TIMP1** | High | 342 | 280-427 | 0.002 | 3.425 | 6e-04 | CAV^high^X^high^ | 142 | 94-342 | 3e-06 | Cav ^high^  =  2.681 | 0.00017 |
|  |  |  |  |  |  |  | CAV^high^X^low^ | - | - |  |  |  |
|  | Low | 989 | 427-NA |  |  |  | CAV^low^X^high^ | 406 | 323-478 |  | TIMP1^high^  =  3.086 | 0.009 |
|  |  |  |  |  |  |  | CAV^low^X^low^ | 989 | 427-NA |  |  |  |
| **TIMP3** | High | 480 | 406-NA | 0.02 |  |  | CAV^high^X^high^ | NA | NA-NA | 8e-06 | Cav ^high^  =  2.825 | 7.4e-05 |
|  |  |  |  |  |  |  | CAV^high^X^low^ | 138 | 87-342 |  |  |  |
|  | Low | 357 | 313-427 |  | 0.508 | 0.01 | CAV^low^X^high^ | 480 | 406-NA |  | TIMP3 ^high^  =  0.544 | 0.04 |
|  |  |  |  |  |  |  | CAV^low^X^low^ | 414 | 342-485 |  |  |  |
| **PAI1** | High | 231 | 146-485 | 0.03 | 1.715 | 0.04 | CAV^high^X^high^ | 150 | 94-NA | 2e-05 | Cav^high^  =  3.215 | 0.00097 |
|  |  |  |  |  |  |  | CAV^high^X^low^ | 106 | 82-NA |  |  |  |
|  | Low | 419 | 359-480 |  |  |  | CAV^low^X^high^ | 313 | 164-NA |  | PAI1^high^  =  1.110 | 0.75732 |
|  |  |  |  |  |  |  | CAV^low^X^low^ | 427 | 360-489 |  |  |  |
| **TSP1** | High | 323 | 270-406 | 0.003 | 2.597 | 0.001 | CAV^high^X^high^ | 142 | 94-342 | 5e-06 | Cav ^high^  =  2.268 | 0.016 |
|  |  |  |  |  |  |  | CAV^high^X^low^ | - | - |  |  |  |
|  | Low | 648 | 439-NA |  |  |  | CAV^low^X^high^ | 359 | 313-478 |  | TSP1^high^  =  2.268 | 0.016 |
|  |  |  |  |  |  |  | CAV^low^X^low^ | 648 | 439-NA |  |  |  |
| **VIM** | High | 342 | 279-427 | 0.2 |  |  | CAV^high^X^high^ | 142 | 94-342 | 6e-05 | Cav ^high^  =  2.890 | 0.00011 |
|  |  |  |  |  |  |  | CAV^high^X^low^ | - | - |  |  |  |
|  | Low | 468 | 414-737 |  | 1.328 | 0.2 | CAV^low^X^high^ | 399 | 323-485 |  | VIM ^high^  =  1.094 | 0.703 |
|  |  |  |  |  |  |  | CAV^low^X^low^ | 468 | 414-737 |  |  |  |
